# Balancing integrated green-grey infrastructure shapes carbon emissions in village-town clusters

**DOI:** 10.1016/j.isci.2025.113902

**Published:** 2025-10-30

**Authors:** Zhuo-Yang Sun, Xiaoqing Zhu

**Affiliations:** 1 College of Civil Engineering, Zhejiang University of Technology, Hangzhou 310058, P.R. China; 2 School of Design and Architecture, Zhejiang University of Technology, Hangzhou 310058, P.R. China; 3 Center For Urban Governance Studies, Hangzhou International Urbanology Research Center, Hangzhou 311121, P.R. China

**Keywords:** environmental science, applied sciences, social sciences

## Abstract

The rapid expansion of built-up land has disrupted carbon dynamics, intensifying climate instability. Understanding how green and gray infrastructure patterns influence carbon emissions is crucial, yet most analyses either treat them separately or overlook local heterogeneity. This study adopts village-town clusters as minimum planning entities to explore how integrated green-grey infrastructure land cover patterns (IGGI-LCPs) affect net carbon emission intensity (NCEI). Using eight representative village-town clusters (74 villages) in Zhejiang Province, China, we developed an IGGI-LCP quantification system and a dual carbon accounting framework. GWR and MGWR show that dense gray infrastructure consistently increases emissions, while green coverage mitigates emissions only when forming large ecological cores. GDP density drives emissions, whereas population density shows indirect, context-specific effects. Threshold analysis shows NDGG ≤ −0.20 for carbon sinks, NDGG ≥ +0.20 for carbon sources, and −0.20 ≤ NDGG ≤ +0.20 indicates a balanced green-grey configuration. Findings emphasize adaptive and region-specific strategies to support low-carbon planning amid rural transformations.

## Highlights

• Introduces an IGGI-LCP framework to quantify green-grey balance in village-town clusters
• Identifies thresholds for balanced emissions using NDGG (−0.20 ≤ NDGG ≤ +0.20)
• Economic density drives emissions, while large ecological cores mitigate them
• Proposes differentiated low-carbon planning strategies based on regional characteristics

## Graphical abstract

**Figure:**
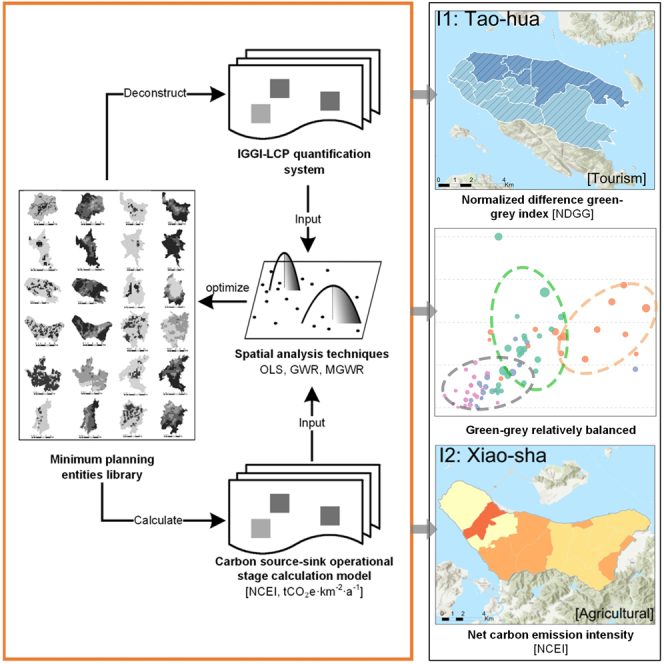

## Introduction

Population growth, resource overutilization, and climate change are among the foremost challenges to sustainable development in the 21st century.^1^ To meet the diverse needs of an ever-growing global population, infrastructure development is accelerating at both global and local scales, fundamentally altering land surface cover.^2^ This rapid expansion creates a competitive dynamic between green and gray surfaces, where development frequently substitutes green infrastructure (GI) with gray infrastructure (GRI)—a shift that contributes to climate change, depletes natural resources, and leads to more frequent extreme weather events.^3^^,^^4^^,^^5^ Approximately 79% (50 billion tons) of global greenhouse gas emissions are attributable to the construction and operation of infrastructure—encompassing power, transportation, and building systems—and contribute to 88% (USD 81.6 billion) of the estimated global costs of climate adaptation.^6^ As global infrastructure investment is projected to continue its upward trajectory over the next two decades,^7^ there emerges both a challenge and an opportunity to reconfigure these systems in a manner aligned with carbon neutrality goals. Understanding how the land cover patterns and spatial configurations of infrastructure influence carbon emissions is essential for developing evidence-based adjustments to infrastructure, ultimately promoting environmental sustainability.^8^^,^^9^^,^^10^^,^^11^^,^^12^

Over the years, studies on green infrastructure (GI) and gray infrastructure (GRI) have generally been conducted in isolation, often focusing on the role of a single category in influencing carbon emissions or sinks, rather than looking at their interactive impacts in shared urban-rural spaces. On the one hand, existing GI research has emphasized vegetation systems—such as trees, shrubs, and grasses—regarding their carbon sequestration potential and ecosystem service provision.^13^^,^^14^^,^^15^ Applying models such as CASA and InVEST, such work has quantified vegetation net primary productivity (NPP) and carbon storage at large scales,^15^^,^^16^ highlighting how extensive green coverage mitigates urban heat islands and reduces energy consumption for cooling. Although some studies discuss how optimized GI patterns can help lower greenhouse-gas emissions,^17^^,^^18^ few focus on how adjacent or expanding GRI can undermine or constrain the actual effectiveness of such GI solutions. On the other hand, GRI research primarily centers on the “carbon source” dimension of infrastructure development, such as embodied carbon in building materials, construction-phase emissions, and the macro-level contributions to rising carbon output due to industrialization and urbanization.^19^^,^^20^^,^^21^^,^^22^^,^^23^ Despite demonstrating how GRI clusters can intensify the heat-island effect and reshape local atmospheric systems—further fueling carbon emissions—existing work lacks a systematic understanding of how GRI interacts with GI within the same spatial domain. Consequently, both fields tend to overlook the reciprocal or synergistic effects of GI and GRI on regional carbon balances, leading to fragmented perspectives that are insufficient to drive genuine progress toward carbon neutrality.

Scholars have thus called for the Integrated Green-Grey Infrastructure (IGGI) paradigm, which aims to investigate GI and GRI within a cohesive system so as to more accurately evaluate their net impact on carbon emissions and environmental sustainability.^24^^,^^25^^,^^26^^,^^27^ IGGI is a comprehensive approach to infrastructure spatial configuration, emphasizing the interconnection and collaboration between ecological systems (natural or semi-natural), engineered systems, and eco-engineered hybrid infrastructures. This integrated framework provides holistic functional benefits within specific socio-economic, environmental, and technical contexts. The evolution of infrastructure paradigms—from the “structure-function” of traditional infrastructure, to the “ecology-network” of green infrastructure, and now the “ecological-social-technical” community model—clarifies the logic behind IGGI’s development. Unlike traditional GI and GRI frameworks, IGGI uniquely integrates landscape ecology (to identify ecological cores and corridors), carbon accounting (to balance sources and sinks across life cycle stages), and spatial economics (to account for factors such as population density, economic output, and land value). In this systemic view, green and gray infrastructures are no longer seen as opposing elements but as complementary, mutually reinforcing components within urban and rural social-ecological systems (SESs), enhancing ecosystem service capacity and environmental adaptability through functional compatibility and system synergy. This integration facilitates the analysis of both competitive interactions (e.g., gray infrastructure replacing green) and complementary synergies (e.g., green buffers reducing emissions from adjacent gray structures) across multiple spatial scales.

Recent studies have made initial progress by jointly modeling processes of GI loss and GRI expansion e.g.,^3^^,^^28^^,^^29^ and, from a microclimatic perspective, by exploring how an optimal spatial configuration of green, gray, and blue infrastructure can reduce regional energy demands and emissions.^30^ Some have also incorporated GI’s carbon-sequestration potential into disaster insurance frameworks.^31^ However, these efforts—while innovative—still lack a robust grasp of the deeper interaction mechanisms and spatial heterogeneity among GI and GRI, as well as insights into how various socioeconomic drivers shape these coupled systems.

In summary, existing research faces four main shortcomings and research gaps. First, it lacks a holistic understanding and quantitative assessment of how green and gray infrastructure, viewed together, influence carbon dynamics, particularly in terms of mitigation potential and emissions patterns. Second, most studies emphasize the construction phase of infrastructure, often overlooking the operational phase, which accounts for a median of 75.2% of life cycle emissions.^32^ Although some argue that spatial layouts minimally influence operational emissions, recent findings suggest that infrastructure distribution impacts energy demand, resource allocation, and local microclimates, ultimately altering emission levels.^33^ Third, most analyses overlook finer spatial units such as villages and towns, losing context-specific insights crucial for localized carbon management. Fourth, the complex interplay among ecological processes, industrial structure, and socioeconomic forces is insufficiently captured, limiting the potential for robust, system-level recommendations to inform carbon neutrality initiatives and sustainable urban development. Only by advancing IGGI conceptual frameworks and employing interdisciplinary approaches can we fully elucidate both the “collaborative” and “competitive” effects of GI and GRI on carbon pathways, thereby providing timely and evidence-based guidance for policymakers and planners.

To address these gaps, this study proposes to use the village-town cluster—the smallest practical planning entity in China—as the focal scale to analyze how integrated green-grey infrastructure land cover patterns (IGGI-LCPs) influence net carbon emission intensity (NCEI) during the operational phase of infrastructure. By selecting eight village-town clusters (74 villages) in Zhejiang Province, we construct an IGGI-LCP quantification system featuring multiple indicators that characterize the spatial distribution and interplay of green and gray infrastructure. In tandem, we develop a dual carbon accounting framework—encompassing both carbon sources and sinks—to map out how operational energy use, transport patterns, and vegetation coverage collectively shape local emissions. Our key contributions are 3-fold: (1) we prioritize village-town clusters over broad-scale county- or city-level analyses, addressing the practical needs of local government planning; (2) we adopt spatial regression methods (GWR/MGWR) to detect non-stationary effects across heterogeneous, rural-urban landscapes, highlighting how region-specific interventions can better mitigate carbon emissions; and (3) we compile a robust operational-phase carbon inventory through extensive field surveys and remote sensing to provide granular insights into rural emission patterns. Ultimately, we aim to advance the IGGI research agenda by clarifying how fine-scale infrastructure configurations can support integrated, context-sensitive carbon reduction strategies, thus offering novel directions for climate-adaptive, low-carbon rural development.

By integrating village-level operational-phase carbon accounting with MGWR analysis, this study reveals pronounced spatial heterogeneity in how green and gray land-cover patterns relate to carbon emissions. Dense gray infrastructure consistently increases NCEI, whereas green infrastructure mitigates emissions only when it forms large, connected ecological cores. GDP density is a strong driver of emissions, while population density exhibits indirect, context-specific effects. We identify a balanced green-grey range (−0.20 ≤ Normalized Difference Green-Grey Index (NDGG) ≤ +0.20) within which most villages maintain moderate emissions, and propose differentiated planning strategies to guide low-carbon rural development. These findings underscore the need for context-sensitive, multi-scale approaches to infrastructure planning and contribute empirical evidence to the evolving IGGI paradigm.

### Study area and data source

#### Study area

China is projected to account for nearly 30% of global infrastructure investments.^7^ Despite the fact that China’s territorial spatial planning is organized at the county level, its fundamental implementation unit is the village-town cluster (a fully established town or a township overseeing multiple villages). Incorporating rural and natural landscapes into regional planning facilitates the context-specific deployment of green and gray infrastructure. This approach is particularly relevant in China, where administrative areas are significantly larger than their built-up areas, providing governments with greater latitude in planning the distribution of carbon sources and sinks. Zhejiang Province (118°01′–123°10' E and 27°02′–31°11' N) exemplifies this trend. As one of the China’s most urbanized and economically dynamic regions, Zhejiang has shifted its developmental focus to rural areas, where per capita carbon emissions have risen 2.4 times faster than in urban counterparts.^34^^,^^35^ This rapid rural transition, coupled with Zhejiang’s diverse geomorphology—ranging from coastal plains to mountainous terrain and islands—makes it an ideal case for examining the interplay between integrated green-grey infrastructure land cover patterns (IGGI-LCP) and carbon dynamics.^36^

**Figure 1:**
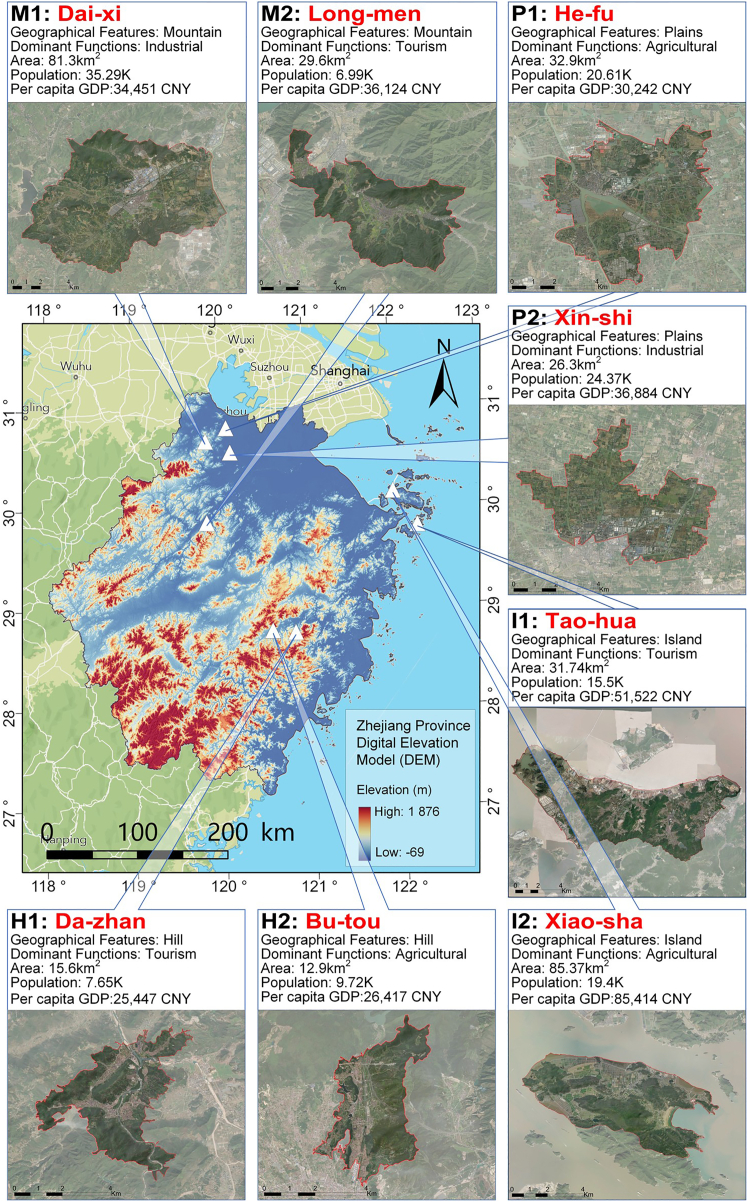
Study area of eight village-town clusters across varied geomorphological types in Zhejiang Province Note: Eight representative village-town clusters (74 villages) were selected from the sample database, covering most of Zhejiang’s topographic features, including mountains, hills, plains, and islands. Each cluster has distinct socio-economic characteristics and dominant industries. (For more details, such as P1 having extensive photovoltaic agro-fishery, please refer to Appendix 1.).

To capture this variability, we selected eight village-town clusters across four geomorphological types (mountain, island, plains, hill) and distinct economic activities (e.g., industry, agriculture, tourism) (Figure 1). This diverse cross-section enables a fine-grained analysis of spatial heterogeneity in emissions, thereby illustrating how green and gray infrastructure patterns intertwine with carbon dynamics in rapidly transitioning rural regions. For further details, please refer to Data S1.

To clarify the selection criteria and representativeness of the eight village-town clusters, we note that each cluster encompasses a unique combination of geomorphology, land-use patterns, and socio-economic function, collectively covering the spectrum of rural landscapes in Zhejiang Province. Mountainous clusters (M1 and M2) primarily feature forestry and eco-tourism with limited industrial activity; hill clusters (H1 and H2) mix agriculture, tourism, and small-scale processing; plains clusters (P1 and P2) represent high-density, industrialized agricultural hubs with intensive energy consumption; and island clusters (I1 and I2) capture maritime economic activities such as fisheries and coastal tourism. By integrating all four landform types and diverse economic modes, our sample provides a holistic microcosm of Zhejiang’s rural transformation. Moreover, many rural planning units in China share similar sizes and functions, enhancing the external validity of our findings.

#### Data sources and processing

Sampling investigations and household questionnaires were administered across 74 villages from January to March and June to September 2022. Based on^37^ and assuming a standard deviation of 0.5 with a 90% confidence level, a sample size of 68 was required for a population of 1,000, allowing for a margin of error of approximately 0.085. To account for varying survey conditions across villages, we increased the number of distributed questionnaires to maintain the 90% confidence level and enhance data reliability. As presented in Table 1, questionnaire sampling rates ranged from 13.0% to 17.1%, with a response rate exceeding 95.7%.

**Table 1:** Sample sizes, response rates, and demographic coverage of the questionnaire survey across eight village-town clusters

Geomorphological Types	Village-town Cluster Identifier [Name]	Total Population [People]	Number of Questionnaires [Count]	Valid Questionnaires [Count]	Population Covered by Valid Questionnaires [People]	Sampling Rate [%]	Validity Rate [%]
Plains	P1 [He-fu]	20613	342	332	3217	15.6%	97.1%
	P2 [Xin-shi]	24372	418	410	3830	15.7%	98.1%
Hill	H1 [Da-zhan]	7653	130	125	1308	17.1%	96.2%
	H2 [Bu-tou]	9723	167	160	1663	17.1%	95.8%
Island	I1 [Tao-hua]	15482	267	260	2574	16.6%	97.4%
	I2 [Xiao-sha]	19377	300	290	2907	15.0%	96.7%
Mountain	M1 [Dai-xi]	35299	460	440	4589	13.0%	95.7%
	M2 [Long-men]	6991	98	94	950	13.6%	95.9%

Energy activity data for each village-town cluster were obtained from sampling investigations, while electricity data were sourced from statistical results provided by the Zhejiang branch of the State Grid Corporation. During the survey, to aid residents' understanding, energy consumption values were converted into more relatable parameters, such as costs (e.g., electricity bills) and weights (e.g., coal mass). Final energy usage data for diverse types were calculated using conversion formulas (e.g., oil consumption = oil cost/oil price × oil density).

A Monte Carlo simulation (±10% variability in activity data; ±5% variability in emission factors) produced distributions of carbon emissions; electricity and coal consumption contributed over 60% of the variance. The median relative width of the 95% confidence interval for NCEI across villages is 43.9% (IQR 43.3%–44.3%), indicating a typical uncertainty of ±22%. Carbon sink data were derived using Sentinel-2 remote sensing imagery as the primary data source, with supplementary use of Landsat data for Normalized Difference Vegetation Index (NDVI) calculations, the MODIS data for vegetation coverage type, and the TerraClimate global monthly climate dataset for meteorological data. The CASA model was applied to estimate vegetation carbon absorption. All satellite data underwent geometric correction, atmospheric correction, cloud removal, and reprojection to ensure accuracy. Data processing was conducted using the Google Earth Engine (GEE) cloud platform, covering January to December 2022 (see Methods S1 for details). Table S1 summarizes all data sources.

## Results

### Descriptive analysis of integrated green-grey infrastructure and carbon emissions patterns

**Figure 2:**
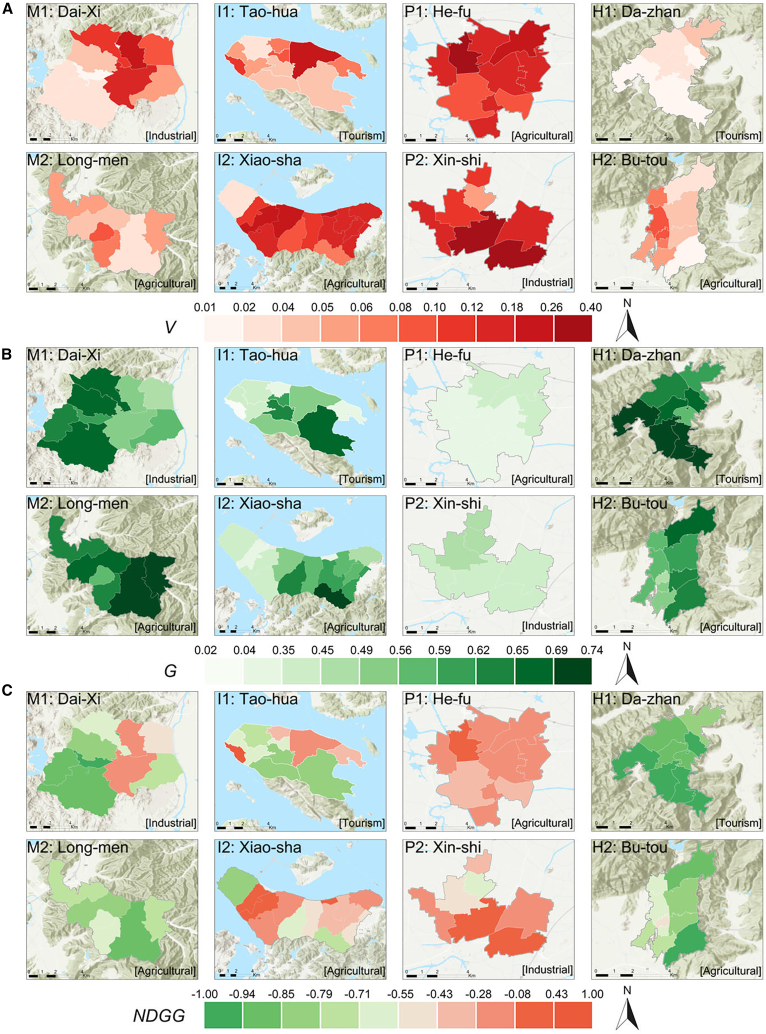
Integrated green-grey infrastructure land cover patterns (gray coverage, green coverage, and NDGG) across the eight village-town clusters (A) Gray coverage ratio (V). (B) Green coverage ratio (G). (C) Normalized difference green-grey index (NDGG).

**Figure 3:**
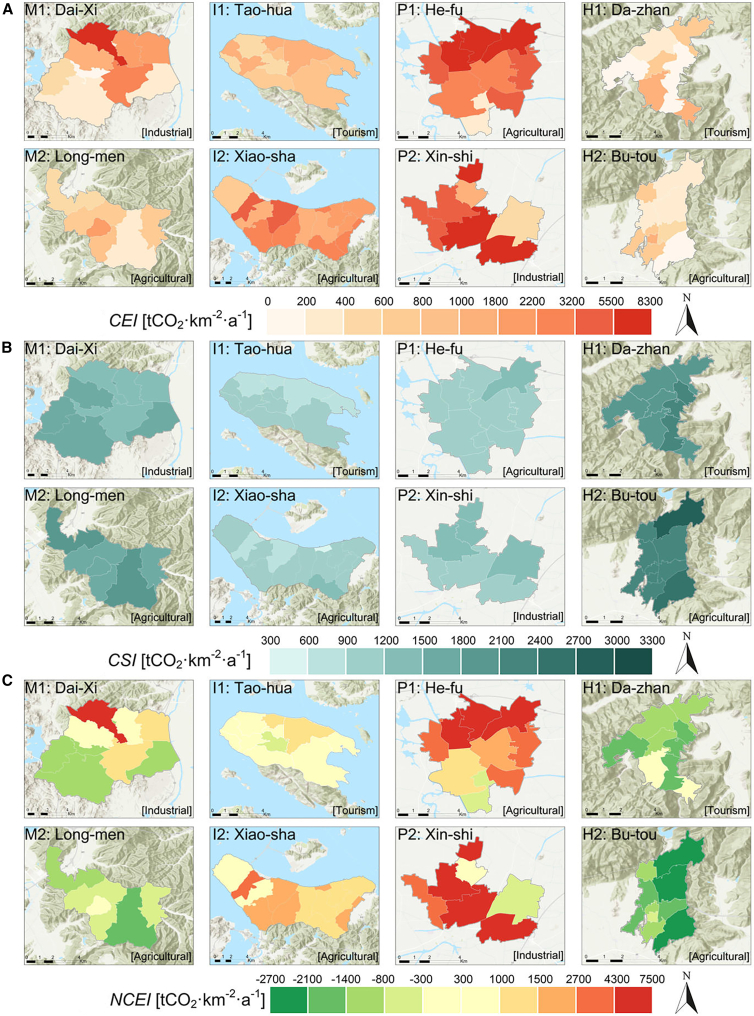
Spatial distribution of carbon emissions, carbon sinks, and net carbon emission intensity across the eight village-town clusters (A) Carbon emission intensity (CEI). (B) Carbon sink intensity (CSI). (C) Net carbon emission intensity (NCEI).

To elucidate the baseline spatial differentiation prior to the regression analysis, we first mapped the integrated green-grey land cover patterns—including V, G, and NDGG—in Figures 2A–2C, together with the carbon source-sink profiles (CEI, CSI, and NCEI) shown in Figures 3A–3C. Overall, the mountain clusters (M1, M2) and hill clusters (H1, H2) exhibit relatively low gray coverage—in many sub-regions, V < 0.06—and comparatively high green coverage (G > 0.60). Such vegetation-rich landscapes commonly yield negative or near-zero NCEI, indicating local carbon sinks or near-balance between emissions and absorptions. For instance, in Dai-xi (M1), industrial clusters in the central-northern area can push V as high as 0.19 (Figure 2A), producing moderate net emissions on the order of +800 to +1400 t CO_2_·km^−2^·a^−1^ (Figure 3C). However, adjacent patches with G > 0.60 effectively offset those emissions, sometimes bringing NCEI closer to zero or even mildly negative (e.g., −200 to −400 t CO_2_·km^−2^·a^−1^). Similarly, Long-men (M2) retains significant vegetation in its central heritage zones, keeping overall NCEI at modest positive or negative levels (within ±500 t CO_2_·km^−2^·a^−1^), despite slightly denser development near its periphery.

By contrast, plains clusters (P1 and P2), where farmland and industry often intersect, feature larger gray footprints—with V occasionally exceeding 0.15 or even 0.20—and limited green coverage (frequently below 0.40) in Figure 2B. As a result, some sub-areas in He-fu (P1) and Xin-shi (P2) register substantially elevated net carbon emissions, sometimes above +3000 or +4000 t CO_2_·km^−2^·a^−1^. This is especially evident in industrial blocks or high-density residential clusters, where built-up surfaces outcompete vegetative land. Meanwhile, island clusters (I1, I2) occupy an intermediate spectrum. In Tao-hua (I1), scenic resources and coastal restrictions help maintain significant vegetation, with many patches displaying NCEI values between −200 and +200 t CO_2_·km^−2^·a^−1^. Conversely, in Xiao-sha (I2), aquaculture-related development and small harbors can push V toward 0.10–0.15, driving local emissions into the +500 to +1000 t CO_2_·km^−2^·a^−1^ range, although adjacent vegetated areas remain near carbon-neutral. Lastly, the hill clusters—Da-zhan (H1) and Bu-tou (H2)—also demonstrate varied grey-green mosaics: while Da-zhan’s hot spring resorts or Bu-tou’s small processing facilities may create localized emission hotspots, extensive hillside vegetation keeps many sub-regions at moderate or near-zero net intensities.

As illustrated in Figures 2 and 3, these diverse green-grey configurations directly shape local carbon balances, highlighting how physical geography, economic function, and land-use management jointly determine emissions across each village boundary. Mountainous and hilly locales typically sustain lower V and, consequently, near-zero or negative NCEI, whereas plains accommodate denser construction and higher net emissions. Island settings, along with certain agricultural or tourism-focused sub-regions, strike a more moderate balance, interspersing vegetated tracts with localized gray expansions. Collectively, these spatial contrasts inform the subsequent regression analysis in Section 4.2, wherein additional socioeconomic variables—such as population density and GDP output—are integrated to further clarify how IGGI configurations influence net carbon emissions in these multi-functional village contexts.

Figure 2C displays the spatial distribution of the Normalized Difference Green-Grey Index (NDGG). Recall that NDGG values range from −1 to +1: values near −1 indicate a dominance of green coverage over gray infrastructure, values near +1 reflect gray dominance, and values near 0 signify a balanced landscape. In the mountain and hill clusters, NDGG typically falls between −0.5 and −0.2, indicating strong green dominance, which corresponds to the observed low NCEI. In contrast, NDGG values exceeding 0.2 in the industrial subregions of plains clusters signify gray dominance and correlate with high NCEI. Island clusters show NDGG values near −0.1 to +0.1, reflecting a mixed landscape with moderate emissions. This composite index thus succinctly encapsulates the green-grey balance and facilitates comparison across clusters; its association with NCEI (see Section 4.2) underscores the importance of controlling gray expansion and enhancing green coverage. Moreover, by juxtaposing Figures 2A–2C, we observe that NDGG captures the combined effects of V and G: high NDGG corresponds to high V and low G, and vice versa. This integrated perspective justifies our choice of NDGG over separate G and V variables.

**Figure 4:**
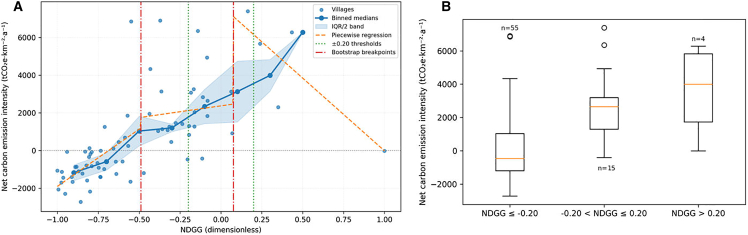
Relationship between the Normalized Difference Green-Grey Index (NDGG) and net carbon emission intensity (NCEI) for all villages (A) ScatterPlot of NDGG vs. NCEI with thresholds. (B) Distribution of NCEI by NDGG band. Note: Scatterplot illustrates the relationship between NDGG and NCEI. Each point represents a village. The dashed vertical lines denote NDGG values of −0.20 and +0.20, highlighting the range where most villages exhibit moderate emissions. The dotted horizontal line indicates NCEI = 0.

Figure 4A further illustrates the relationship between NDGG and NCEI. Each point represents a village. The dashed vertical lines at −0.20 and +0.20 demarcate the planning thresholds; the dot-dash lines mark the bootstrapped breakpoints (approx. −0.489 and +0.076). Binned medians and IQR ribbons summarize the central trend. Villages with NDGG ≤ −0.20 generally have low or negative emissions, while those with NDGG ≥ +0.20 typically have high emissions. Figure 4B groups NCEI by NDGG band (≤−0.20, −0.20 < NDGG ≤ +0.20, > +0.20), showing median NCEI of −470, +2645 and +3988 t CO_2_e·km^−2^·a^−1^, respectively. These observations support using −0.20 ≤ NDGG ≤ +0.20 as a balanced green-grey range. (See method details for details).

### Regression results

#### Stepwise ordinary least-squares results

This section used 3 OLS stepwise models as the basic model to test the relationship between driving factors and NCEI, which is also the classic global regression model. In interpreting the OLS models, we emphasize three key findings: (i) V_util_ consistently exerts a significant positive influence on NCEI across all models, highlighting that vertical and horizontal intensification of gray infrastructure amplifies carbon emissions; (ii) NDGG emerges as a significant positive driver of NCEI, indicating that gray dominance exacerbates emissions, while green dominance mitigates them; (iii) R_core_ shows a negative effect in the baseline model but becomes insignificant when NDGG and socioeconomic variables are added, suggesting that ecological cores alone cannot offset emissions when surrounded by extensive gray infrastructure. Economic density (D_GDP_) significantly increases emissions, whereas population density (D_pop_) remains insignificant, implying that population impacts are indirect. These insights justify the use of spatial models to further dissect heterogeneity. The OLS results in Table 2 show that R^2^ improves progressively from 0.438 (OLS 1) to 0.566 (OLS 3). The Adj. R^2^ also improves consistently, indicating that the inclusion of additional variables significantly enhances the models' ability to explain the variation in NCEI. The variance inflation (VIF) for all variables remains below 5, ranging from a minimum of 1.278 to a maximum of 2.615. Therefore, the assumption that there is multicollinearity among independent variables is rejected, indicating no redundancy in the variables.

**Table 2:** Stepwise ordinary least-squares (OLS) regression results linking integrated green–grey infrastructure variables to NCEI

Model	Variables	Coefficients	Significance	*p*-value	VIF
OLS 1 R^2^ = 0.438 Adj.R^2^ = 0.422	ln(V_util_)	0.306	∗∗∗	0.001	1.278
	R_core_	−0.375	∗∗∗	0.001	1.278
	Constant	0.425	–	–	–
OLS 2 R^2^ = 0.525 Adj.R^2^ = 0.505	ln(V_util_)	0.280	∗∗	0.003	1.283
	R_core_	−0.159	–	0.105	2.084
	NDGG	0.479	∗∗∗	0.001	1.870
	Constant	0.210	–	–	–
OLS 3 R^2^ = 0.566 Adj.R^2^ = 0.535	ln(V_util_)	0.257	∗∗∗	0.005	1.303
	R_core_	−0.197	–	0.051	2.321
	NDGG	0.274	–	0.079	2.615
	ln(D_GDP_)	0.329	∗	0.013	2.533
	ln(D_pop_)	−0.145	–	0.218	1.966
	Constant	0.186	–	–	–

Note: ∗∗∗ Represents statistically significant at the *p* < 0.001 level, ∗∗ Represents statistically significant at the *p* < 0.01 level, Represents statistically significant at the ∗*p* < 0.05 level.

Coefficient estimates of independent variables are consistent with the research hypothesis in terms of signs and magnitudes. However, the significance levels (*p*-values) of some variables change across the stepwise regression process. Specifically, as new variables are introduced, the significance of previously included variables diminishes, reflecting the shared explanatory power among them. While OLS models provide an initial understanding of the relationships between variables and NCEI, their assumption of global spatial stationarity limits their ability to capture localized variations, potentially overlooking critical spatial heterogeneity within the data. The results show that V_util_ consistently exerts a significant positive influence across all three models, emphasizing that the vertical development of infrastructure, rather than just its horizontal expansion, significantly intensifies regional carbon emissions. R_core_ demonstrates a significant negative effect in OLS 1, highlighting the carbon absorption capacity of ecological zones. However, its significance diminishes as the NDGG and socioeconomic variables are introduced, indicating that part of its explanatory power is shared with the newly added variables. NDGG emerges as a positive driver of NCEI, suggesting that higher proportions of gray cover exacerbate carbon emissions. Additionally, ln(D_GDP_) becomes significant in OLS 3, underscoring the role of economic activity, while ln(D_pop_) remains statistically insignificant across all models, implying an indirect or spatially heterogeneous effect.

**Figure 5:**
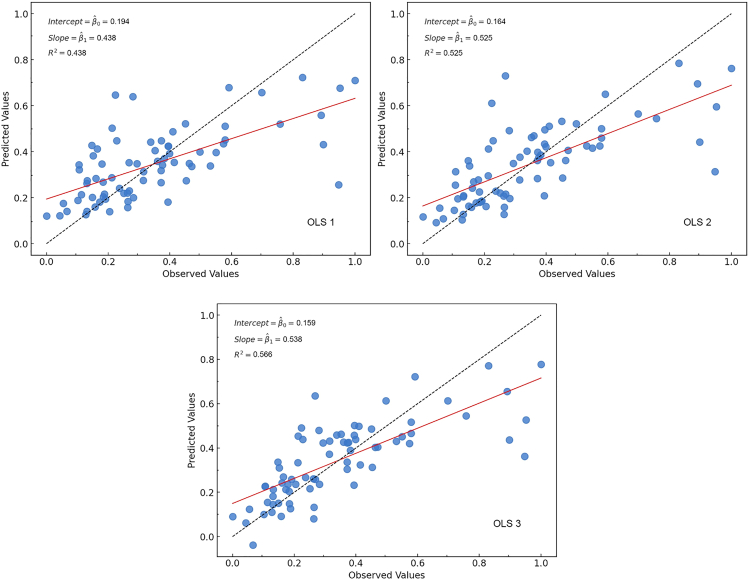
Comparison of observed versus predicted NCEI values across stepwise OLS models

The scatter diagrams in Figure 5 illustrate the models’ fitting performance. OLS 1 shows a dispersed distribution of points around the 1:1 line, while OLS 3 achieves the best fit, with points clustering more closely and a higher R^2^. This comparison confirms that OLS 3 provides the highest explanatory power and the best fitting performance among the three models. The Moran’s I results in Table 3 reveal changes in the spatial autocorrelation of model residuals as variables are added. In OLS 1, Moran’s I is 0.248 (*p* = 0.021), indicating significant positive spatial autocorrelation and suggesting the baseline model fails to fully capture spatial patterns. After including NDGG in OLS 2, Moran’s I decreases to 0.178 (*p* = 0.037), indicating that NDGG helps reduce spatial dependence by accounting for some spatial patterns related to the balance between gray and green infrastructure. In OLS 3, the inclusion of additional socio-economic variables (ln(D_GDP_) and ln(D_pop_)) further lowers Moran’s I to 0.153 (*p* = 0.073), approaching randomness. This suggests that these variables improve the model’s ability to capture certain spatial variations, though residual spatial dependence is not fully eliminated.

**Table 3:** Spatial autocorrelation (Moran’s I) of the residuals from the OLS models

Model	Moran’s I	*Z* Score	*P*-value
OLS 1	0.248	2.056	0.021
OLS 2	0.178	1.834	0.037
OLS 3	0.153	1.623	0.073

The persistence of weak spatial autocorrelation in OLS 3 highlights the limitations of global models in addressing spatial heterogeneity. While variables such as V_util_ and ln(D_GDP_) exhibit stable global effects, others, such as NDGG and R_core_, may display spatially varying relationships. Therefore, to better address localized variations and spatial heterogeneity, spatial regression models are necessary.

To evaluate NDGG’s predictive value beyond rank stability, we treated NDGG as a single predictor of NCEI and performed both 5-fold cross-validation and leave-one-cluster-out validation. For each fold, we fitted a simple linear regression on the training data and computed the coefficient of determination (R^2^) and mean absolute error (MAE) on the test set. The 5-fold R^2^ scores ranged from −0.27 to 0.51 (mean ≈0.25), with MAE ≈900–1643 t CO_2_e·km^−2^·a^−1^ (Table 4). When entire clusters were held out, R^2^ values were often negative (mean ≈ −5.32) and MAEs remained large, underscoring NDGG’s limited ability to generalize across heterogeneous clusters. These results confirm that NDGG is best interpreted as part of a multivariate framework rather than as a standalone predictor.

**Table 4:** Five-fold cross-validation of NDGG as a stand-alone predictor of net carbon emission intensity

Validation scheme (fold)	R^2^	MAE (t CO_2_e km^−2^ yr^−1^)
Five-fold CV fold 1	0.4877	1252.0
Five-fold CV fold 2	−0.2706	1449.3
Five-fold CV fold 3	0.5085	899.9
Five-fold CV fold 4	0.2906	1101.1
Five-fold CV fold 5	0.2206	1643.2
Mean	0.247	1269.0

Note. In leave-one-cluster-out validation, the mean R^2^ is –5.32 and the mean MAE is 1 390 t CO_2_e km^−2^ yr^−1^.

#### GWR, MGWR results

To address the spatial heterogeneity not fully explained by the OLS models, GWR and MGWR models were applied, offering improvements in model fit and a more precise representation of local effects. The results in Table 5 show that GWR significantly improves model performance, with the R^2^ increasing from 0.566 in OLS 3 to 0.734 in GWR, and the Adjusted R^2^ rising to 0.665. The AICc decreases notably from 163.870 to 153.299, reflecting the improved explanatory power of GWR by allowing spatially varying coefficients. MGWR further optimizes the results, achieving an R^2^ of 0.736, an Adjusted R^2^ of 0.673, and a lower AICc of 150.110. The improvements reflect MGWR’s superior ability to provide more stable and precise estimates of localized impacts. Additionally, the reduction in residual square sums (RSS) from 32.089 in OLS 3 to 19.553 in MGWR underscores its improved explanatory power.

**Table 5:** Comparison of model fit among OLS, geographically weighted regression (GWR), and multiscale GWR (MGWR) models

Model	AICc	R^2^	Adjusted R^2^	RSS	Sigma Estimate
OLS	163.870	0.566	0.535	32.089	–
*GWR*	153.299	0.734	0.665	19.706	0.577
MGWR	150.110	0.736	0.673	19.553	0.571

The local parameter estimates for MGWR (Table 6) reveal distinct spatial heterogeneity. Among the explanatory variables, V_util_ maintains a consistent positive impact across regions, with significant coefficients observed in 43.243% of the study area. This indicates that increased gray infrastructure utilization remains a stable driver of NCEI, particularly in regions with dense urban and industrial infrastructure. In contrast, NDGG, representing the balance between green and gray infrastructure, shows more pronounced spatial variability, with significant positive effects observed in 67.568% of regions. These effects are especially concentrated in industrial and agricultural areas, where a higher proportion of gray infrastructure exacerbates carbon emissions. We observe that when NDGG >0.20, most villages become net carbon sources; when NDGG < −0.20, villages often function as carbon sinks or are nearly carbon-neutral; and when NDGG lies between −0.20 and 0.20, the landscapes tend to achieve a balance. This threshold effect highlights the nonlinear nature of green-grey interactions and provides a quantitative guide for planning.

**Table 6:** Descriptive statistics of MGWR coefficient estimates and proportion of villages with significant effects

GWR Model					MGWR model					Percentage of Villages by Significance (95% Level) of t-Test
Variable	GWR Coefficients				Variable	MGWR Coefficients				
	Mean	Min	Max	STD		Mean	Min	Max	STD	*p* ≤ 0.05 (%)
Intercept	0.048	−0.493	0.309	0.299	Intercept	0.040	−0.451	0.306	0.278	59.459
ln(V_util_)	0.192	0.099	0.283	0.078	ln(V_util_)	0.164	0.032	0.267	0.093	43.243
R_core_	−0.060	−0.482	0.205	0.283	R_core_	−0.034	−0.273	0.141	0.172	0.000
NDGG	0.331	−0.053	0.743	0.302	NDGG	0.321	0.033	0.529	0.209	67.568
ln(D_GDP_)	0.205	0.091	0.250	0.057	ln(D_GDP_)	0.207	0.095	0.354	0.113	43.243
ln(D_pop_)	−0.007	−0.219	0.180	0.139	ln(D_pop_)	−0.008	−0.130	0.108	0.098	0.000
R^2^	0.734	–	–	–	R^2^	0.736	–	–	–	–
Adj.R^2^	0.665	–	–	–	Adj.R^2^	0.673	–	–	–	–
AICc	153.299	–	–	–	AICc	150.11	–	–	–	–

Note: The percentages under “*p* ≤ 0.05 (%)” indicate the proportion of regions where the variable’s coefficient is significant at the 95% confidence level, while “+” and “−” indicate positive and negative significant coefficients, respectively.

R_core_ demonstrates a more localized impact, with significant coefficients absent across all regions. While its average effect is negative, indicating potential mitigation of carbon emissions through green infrastructure, the lack of statistical significance suggests its influence is spatially constrained or overshadowed by dominant gray infrastructure. Similarly, ln(D_GDP_) emerges as a significant driver in 43.24% of regions, reflecting the strong positive correlation between economic activity and NCEI in areas with concentrated economic output. In contrast, ln(D_pop_), representing population density, fails to exhibit significant effects in most regions, underscoring its limited direct influence on carbon emissions.

#### Bandwidth and spatial distribution analysis of regression coefficients

**Figure 6:**
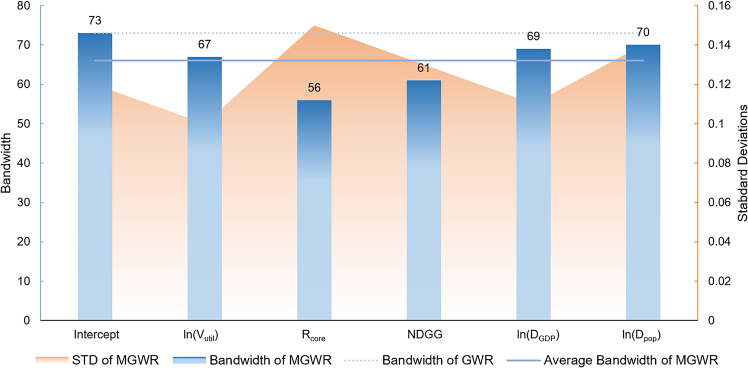
Bandwidth selection results for explanatory variables in the multiscale geographically weighted regression (MGWR) model

Figure 6 presents the bandwidth results for each variable in the MGWR model, reflecting the spatial scale of their impacts. Variables such as R_core_ (56) and NDGG (61) exhibit smaller bandwidths, indicating localized effects and significant spatial heterogeneity. However, as noted in Section 4.2.2, R_core_ lacks statistical significance across most regions, limiting its practical influence despite its spatial variability. By contrast, NDGG shows significant positive effects in 67.57% of regions, particularly in industrial and agriculturally based villages, highlighting its role as a key localized driver of carbon emissions. These findings align with our conceptual distinction between local ecological factors and broader socioeconomic drivers and justify the differentiated strategies proposed in Section 5.

Conversely, ln(V_util_), ln(D_GDP_), and ln(D_pop_) demonstrate larger bandwidth (67–70), suggesting broader spatial scales and relatively stable global patterns. Among these, ln(V_util_), ln(D_GDP_) maintain statistically significant and positive influences over substantial portions of the study area, underscoring their global contributions to NCEI. ln(D_pop_) remains statistically insignificant, reflecting its limited direct impact despite its spatial stability.

**Figure 7:**
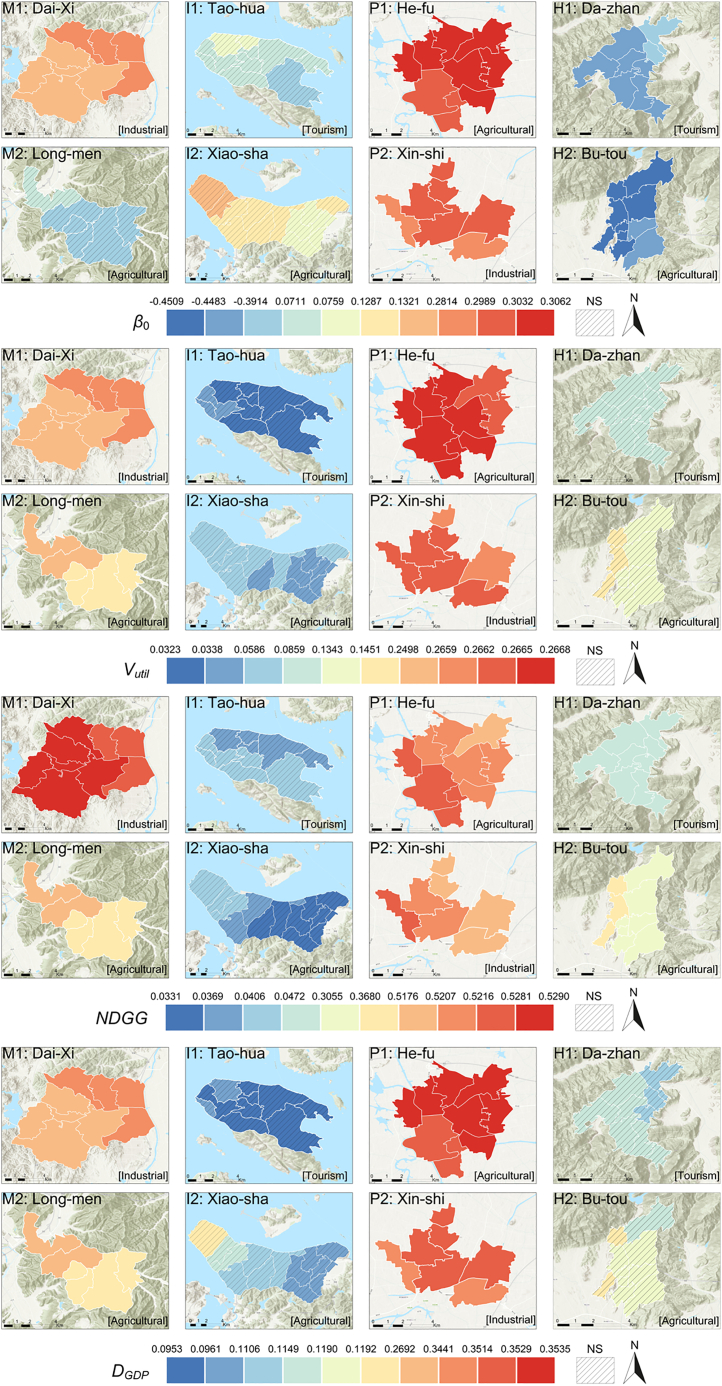
Spatial distribution of local MGWR regression coefficients for the key explanatory variables (NDGG, V_util_ and D_GDP_) Note: Variables (R_core_, ln(D_pop_)) with all regions showing *p* > 0.05 are not displayed.

In the MGWR coefficient spatial distribution analysis (Figure 7), the effects of various variables on NCEI exhibit marked spatial variations across different village-town clusters. MGWR assigns an independent bandwidth and parameter estimates for each location, enabling a more precise depiction of the regional differences between IGGI-LCP and NCEI.

First, NDGG demonstrates pronounced spatial heterogeneity. MGWR results indicate that areas with high positive NDGG coefficients are predominantly found in village-town clusters characterized by intensive infrastructure and high development density. In these areas, the large proportion of gray infrastructure—dense distributions of buildings, roads, and industrial facilities—intensifies the carbon emission effect. Taking the Xin-shi (P2) and the Dai-xi (M1) as examples, both feature high population density and strong industrial clustering (e.g., P2’s robust industrial base and M1’s cosmetics industry chain), making NDGG’s positive influence even more pronounced. This suggests that in regions where population density and industrial concentration are high, the reinforcing effect of gray infrastructure expansion on carbon emissions surpasses the mitigating effect of green vegetation, leading to a notable increase in NCEI. Similarly, V_util_ is positively associated with NCEI in most areas, confirming our previous inference that denser, multi-story built environments concentrate energy consumption and elevate carbon emission intensity.

ln(D_GDP_) also shows notable spatial differences. In economically active areas—such as plains regions dominated by industry such as P2, or areas blending tourism and industry such as M1—the positive effect of ln(D_GDP_) is more evident. Higher GDP density generally correlates with more concentrated production and energy consumption activities, resulting in increased carbon emission intensity. This aligns with the background data: for instance, P2 has a higher per capita GDP and output per unit area, along with frequent industrial clustering and manufacturing activities, making the economic factor’s contribution to NCEI particularly evident in this spatial context.

In contrast, R_core_ and ln(D_pop_) exhibit non-significant or limited effects at the local scale, with relatively stable coefficient distributions. Although R_core_ can help mitigate carbon emissions at an overall scale—given its stronger carbon sequestration and ecological stabilization functions—it does not consistently show significant negative effects at the MGWR local scale. This may be because some ecological core patches exist, but their scale or quality is insufficient to effectively offset the high emissions caused by surrounding gray infrastructure. Similarly, ln(D_pop_) does not exhibit substantial spatial heterogeneity in most areas, or its effects may be more indirect, realized through intermediaries such as industrial structure, economic activity intensity, or land use patterns.

In island village-town clusters (e.g., I1 Tao-hua and I2 Xiao-sha), the influences of NDGG and economic factors differ from inland areas. I1 has abundant ecological resources and high green coverage, while I2 relies on fisheries and agriculture; its terrain and land use lead to a more dispersed distribution of gray infrastructure. As a result, NDGG and ln(D_GDP_)’s impact is weaker than in inland industrial areas. These island regions rely on natural resources and traditional fisheries and agriculture, resulting in a more balanced green-grey infrastructure configuration. This balance makes the NDGG coefficient distribution and its degree of influence differ significantly from that of inland industrial clusters.

**Figure 8:**
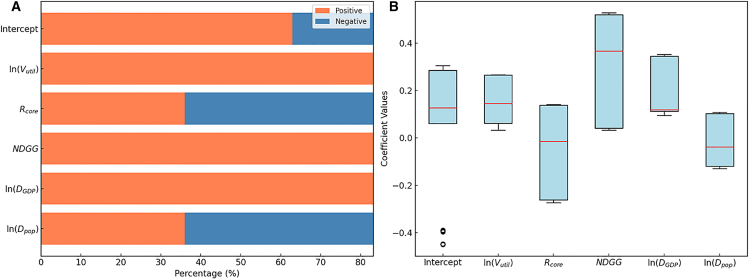
Statistical summary of the direction and magnitude of explanatory-variable effects in the MGWR analysis (A) Proportion of positive and negative effects of different variables. (B) Statistical distribution of different variables’ coefficient estimates.

Figure 8 provides quantitative insights into the direction and magnitude of these effects. Figure 8A shows the proportion of positive and negative impacts for each variable. V_util_ and NDGG exhibit predominantly positive effects across most regions, and ln(D_GDP_) is also mainly positive, supporting the previous significance findings and confirming these three as principal drivers of carbon emissions. Although R_core_ shows negative effects in some areas, the proportion is insufficient to indicate widespread mitigation potential. ln(D_pop_)’s positive and negative effects vary by region, suggesting indirect or context-dependent influences. Figure 8B presents the statistical distributions of coefficient estimates. V_util_, NDGG, and ln(D_GDP_) cluster in the positive range, reinforcing their strong role in driving emissions upward in certain areas. R_core_ and ln(D_pop_) coefficients are more dispersed, with some regions showing weak positive or negative effects, consistent with the earlier spatial heterogeneity analysis.

In summary, the MGWR results reveal the multiscale spatial dynamics of driving factors affecting NCEI. Variables such as NDGG and R_core_ demonstrate localized effects, as indicated by their smaller bandwidths and spatially heterogeneous coefficients, necessitating targeted regional interventions. In contrast, ln(V_util_) and ln(D_GDP_) operate at broader spatial scales, emphasizing their consistent global influence on carbon emissions. Coefficient maps and statistical analyses together confirm that MGWR effectively captures both local and global impacts, providing more nuanced insights into the spatial dynamics of NCEI.

Our early OLS models included both R_core_ and R_edge_. These variables are highly collinear—R_core_ explains most of the variation that R_edge_ would, and their Pearson correlation is −0.73—so including both inflates variance and impairs the MGWR calibration. We therefore dropped R_edge_ and retained R_core_ in the multivariate models. The MGWR results show that R_core_’s coefficient is not significant across most locations. This likely reflects measurement limitations: R_core_ captures only the area of ecological cores, ignoring fragmentation or connectivity. A sensitivity test using a connectivity index *R*_conn_ = *R*_core_/(*R*_core_ + *R*_edge_) yielded a significant negative effect on NCEI in an OLS model (coefficient ≈ −2036, *p* = 0.012). This suggests that ecological cores do mitigate emissions when their size and connectivity are jointly considered. The lack of significance in MGWR thus reflects measurement limitations and spatial scale mismatch rather than a true absence of ecological effects.

## Discussion

**Figure 9:**
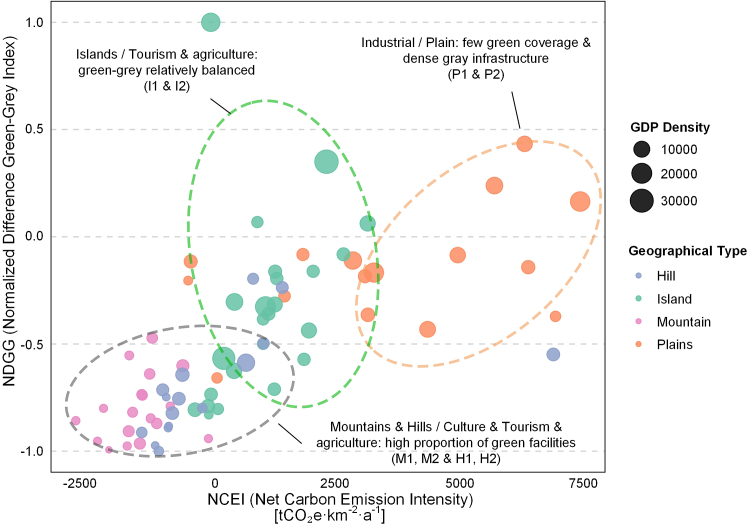
Functional and geographical clustering of village-town clusters based on NDGG and net carbon emission intensity Note: Ellipses indicate representative functional-geographical clusters discussed in the text. Some transitional or hybrid villages are not enclosed due to mixed characteristics. For example, some orange points show lower emissions due to tourism dominance.

This study, using MGWR, explores spatial heterogeneity in the relationship between IGGI-LCP and NCEI across various village-town clusters. The findings show that regional conditions, industrial structure, infrastructure density, and ecological characteristics collectively shape the spatial patterns of carbon emissions. The impacts are neither linear nor uniformly distributed; instead, they differ significantly among village-town clusters with varied functional orientations and geographical features. In industrially concentrated, flat-terrain areas, high gray infrastructure density and intensive economic activity drive higher emissions: the dominance of factories, warehouses, and transport links elevates both direct (energy use) and indirect (land conversion) carbon outputs, while green spaces are often too fragmented to provide meaningful mitigation. Consequently, these regions form a “high-emission, grey-intensive” cluster on the NDGG-NCEI scatterplot, echoing research that impervious surface expansion significantly raises overall emissions^38^ (Figure 9). By contrast, mountainous, hilly, and island clusters exhibit lower economic density and greater ecological coverage, affording them partial insulation from high emissions. In these settings, contiguous green patches—forests, wetlands, agricultural fields—serve as effective carbon sinks, aligning with the global literature on the importance of large, connected green infrastructure.^39^^,^^40^ Even so, the potential of green cover to offset emissions is not boundless: scattered or narrow green corridors in highly industrialized areas showed only marginal impacts on overall emissions.

These findings mirror established relationships between land use, industrial structure, and carbon emissions.^41^^,^^42^ Villages characterized by heavy industry—especially chemical or metal production—display a steeper NDGG-NCEI slope, indicating that each increment of gray infrastructure provokes a disproportionately large emission rise. Meanwhile, tourism- or service-oriented villages, though also on the plains, often maintain moderate emissions and retain greener surroundings. This echoes the broader IPAT framework (Impact = Population × Affluence × Technology), emphasizing that while rising GDP (affluence) typically intensifies emissions, local economic structure and technological choices significantly modify these outcomes. Relatedly, population density alone did not demonstrate a consistent, direct effect on emissions; rather, it interacted with economic factors. A densely populated area built around heavy manufacturing contributes more to emissions than one oriented toward services or eco-tourism. Thus, from a policy standpoint, population and infrastructure considerations should be integrated with the economic base and land-use strategies, rather than using population density as a blanket proxy for emissions potential.

Drawing from the piecewise regression and bootstrap results, we propose a target range of −0.20 ≤ NDGG ≤ +0.20 for achieving a balanced green-grey configuration. Villages with NDGG values below −0.20 generally act as net sinks or exhibit low emissions, while those above +0.20 are associated with higher emissions. These thresholds align with the 95% confidence intervals of the empirically derived breakpoints and approximate the optimal segmentation for planning, with the sum of squared errors (SSEs) within 1% of the minimum.

The MGWR bandwidths reveal insights for planning interventions. NDGG and R_core_, with smaller bandwidths (56–61 villages), indicate that green-grey balance should be managed at the local scale through village-level zoning, vegetated buffers, and community-based greenery programs. In contrast, variables such as Vutil and DGDP, which have larger bandwidths (67–70 villages), operate at broader regional scales, suggesting the need for county-level industrial standards, energy-efficiency codes, and incentives for compact mixed-use development. Recognizing these scales ensures that interventions are appropriately targeted at the relevant administrative levels.

Finally, the Monte Carlo analysis highlights the uncertainty in carbon accounting. For the 74 villages, the median relative width of the 95% confidence interval for NCEI is 43.9% (IQR: 43.3%–44.3%), reflecting ±22% uncertainty around the baseline. This variability stems from ±10% fluctuations in activity data and ±5% in emission factors, with electricity and coal use accounting for more than 60% of the total variance. Planners should thus treat NCEI estimates as ranges, emphasizing the need for improvements in energy-use data collection.

The observed heterogeneity arises from interactions among physical geography, socioeconomic structure, and land-use policy. Mountain and hill villages, with extensive ecological cores and low Vutil, tend to have NDGG ≤ −0.2 and negative or near-zero NCEI. Plains industrial villages show NDGG ≥0.2 due to concentrated factories, warehouses, and transport networks, resulting in high emissions. Island villages fall between these extremes (NDGG ≈ −0.1 to 0.1); coastal protections and fisheries limit gray expansion, while blue-carbon ecosystems and tourism help moderate emissions. Local planning policies and industrial structures further modulate these patterns: tourist villages on plains maintain lower NDGG and NCEI despite economic vibrancy. Understanding these mechanisms underscores the need for tailored interventions rather than one-size-fits-all policies.^43^^,^^44^

Based on MGWR insights and recent literature, we propose quantitative design principles and region-specific strategies to guide low-carbon rural planning.

(1) Mountain & hill villages: Keep NDGG below −0.3 by restricting new gray infrastructure and conserving large, connected ecological cores (R_core_ ≥ 60%). Encourage eco-tourism and sustainable forestry; retrofit existing buildings for energy efficiency instead of expanding their footprint; maintain green corridors and protect watershed vegetation to sustain carbon sinks.
(2) Plain industrial villages: Maintain NDGG within −0.2 ≤ NDGG ≤0.2. When NDGG exceeds 0.2 or falls below −0.2, consider moratoriums on further gray expansion and require grey-to-green compensation (e.g., rooftop gardens, permeable pavements). Promote industrial upgrading and energy-efficient technologies; develop vertical mixed-use buildings to reduce land take while improving Vutil; and introduce linear green spaces between industrial blocks to mitigate emissions.
(3) Island villages: Keep NDGG between −0.1 and 0.1 by preserving coastal wetlands, mangroves, and other blue-carbon ecosystems. Strictly control shoreline development and aquaculture expansion; incentivize renewable energy for transport and fishing fleets; and develop low-impact tourism that complements conservation.

These strategies should be embedded in local land-use plans and carbon management frameworks. We advocate establishing an IGGI-based zoning and assessment system with NDGG and R_core_ thresholds as planning criteria, so that local authorities can approve or reject projects based on their alignment with green-grey balance and carbon neutrality goals. It is important to note that the −0.20 ≤ NDGG ≤ +0.20 band is not the statistically “optimal” break; rather, it is a symmetric and easily interpretable interval that falls within the data-driven breakpoints’ confidence intervals. This symmetry facilitates land-use zoning and communication while remaining consistent with the underlying data. Meanwhile, the ROC-derived threshold (≈−0.587) is asymmetrical and suited to classifying net sources versus net sinks, not to defining balanced green-grey landscapes. Recognizing this distinction helps planners choose thresholds appropriate to their objectives.

### Limitations of the study

Despite the contributions of this study, several limitations should be acknowledged. First, the study focuses exclusively on operational-phase emissions (Scopes 1 and 2) and does not account for embodied carbon from infrastructure lifecycles, which may significantly affect the total carbon footprint. Second, the analysis uses annual average data, which may mask seasonal variations in carbon uptake and energy demand, potentially affecting the accuracy of the estimates. Third, the study is based on a regional case in Zhejiang Province, which limits its generalizability to other regions with different climatic, socioeconomic, and policy contexts. Lastly, the NDGG metric is two-dimensional and does not capture vertical structures, which could be important in urbanized areas. Future studies should integrate LiDAR data to develop three-dimensional NDGG metrics and further explore the seasonal and embodied carbon aspects to enhance the robustness of the analysis.

## Resource availability

### Lead contact

Further information and requests for resources and reagents should be directed to and will be fulfilled by the lead contact, Xiaoqing Zhu (zxq@zjut.edu.cn).

### Materials availability

This study did not generate any new physical materials.

### Data and code availability

• Data: This article uses existing publicly available data. These datasets are listed in the key resources table.
• Code: All data and code supporting the findings of this study are available at our GitHub repository: https://github.com/sunzhuoyang/iggi-data-code. Data S1 contains summary socio-economic and ecological indicators for the eight village-town clusters. The CASA model implementation (Methods S1) is available in the same repository as `casa_model_code.js`.
• All other requests: Any additional information required to reanalyze the data reported will be shared by the lead contact upon request.

## Acknowledgments

We would like to thank the editors and reviewers for their affirmation and help in improving the quality of this article. This work was supported by the 10.13039/501100001809National Natural Science Foundation of China (Grant No. 52578103), the 10.13039/501100012456National Social Science Fund of China (Grant No. 24FJYB035), the Zhejiang Social Science Empowerment Action Special Project (2024-155), the Zhejiang Provincial Department of Housing and Urban-Rural Development Standard Science and Technology Special Project (ZJZX-202108116), the Basic Research Business Fund for Zhejiang Provincial Universities (GB202301005), and the Zhoushan Social Science Empowerment Action Special Project (25FNXD001ZD).

## Author contributions

Conceptualization, Z.S. and X.Z.; methodology, Z.S.; investigation, Z.S.; writing—original draft, Z.S. and X.Z.; writing—review and editing, Z.S. and X.Z.; funding acquisition, X.Z.; resources, Z.S. and X.Z.; supervision, X.Z.

## Declaration of interests

The authors declare no competing interest.

## STAR★Methods

### Key resources table

**Table:** 

REAGENT or RESOURCE	SOURCE	IDENTIFIER
**Deposited data**		

Sentinel-2 (COPERNICUS/S2_SR_HARMONIZED)	European Space Agency (ESA)	https://www.esa.int/Applications/Observing_the_Earth/Copernicus/Sentinel-2
Landsat (9, 8 OLI and TIRS, 7)	United States Geological Survey (USGS)	https://www.usgs.gov/landsat-missions/landsat-9
TerraClimate Global Monthly Climate Dataset (IDAHO_EPSCOR/TERRACLIMATE)	University of California Merced	https://www.climatologylab.org/terraclimate.html
MODIS Vegetation Cover Type (MOD12Q1)	United States Geological Survey (USGS)	https://modis.gsfc.nasa.gov/data/dataprod/mod12.php
Data S1. Summary of socio-economic and ecological indicators for eight village-town clusters	This manuscript	https://github.com/sunzhuoyang/iggi-data-code
Methods S1. CASA model implementation code for carbon-sink estimation	This manuscript	https://github.com/sunzhuoyang/iggi-data-code

**Software and algorithms**		

ArcGIS	ESRI	https://www.arcgis.com/index.html

### Experimental model and study participant details

This article does not contain any studies with all the experimental models (animals, human subjects, plants, microbe strains, cell lines, primary cell cultures) performed by any of the authors.

### Method details

#### Methodology

Figure S1 presents the overview of the methodological framework. We begin by considering multiple factors influencing green and grey infrastructure—such as structural quality, density, and their relative differences—to develop an IGGI-LCP indicator system for detailed spatial analysis. Next, we focus on the operational stage of infrastructure, prioritizing operational carbon metrics over embodied carbon to better capture carbon emissions during usage. To overcome data limitations in small-scale areas (e.g., villages), which are often inadequately served by traditional top-down methods, we adopted a bottom-up data collection strategy. This included field surveys and household questionnaires to gather energy activity data. Additionally, we collaborated with local government bodies to obtain maps of infrastructure footprints, GDP statistics, and data on geographical features and dominant functions. Advanced spatial analysis techniques, including Geographically Weighted Regression (GWR) and Multiscale GWR, were employed to capture spatial non-stationarity and reveal the spatial heterogeneity between IGGI-LCP and carbon sinks & sources, thus supporting the research objectives. The details of the methodology will be discussed in subsequent chapters.

##### Calculating net carbon emission intensity

In this section, we present a dual-model methodology for calculating NCEI that combines a bottom-up model for carbon emissions estimation, capturing both direct and indirect energy consumption from grey infrastructure (e.g., residential and commercial buildings, transportation systems, and public facilities), and a top-down model for carbon absorption quantification, focusing on green infrastructure (e.g., forests, grasslands, and agricultural lands) through NPP calculations derived from remote sensing and field data. The bottom-up model utilizes data from field surveys and infrastructure energy usage records to ensure the accurate quantification of localized carbon emissions. Meanwhile, the top-down model leverages high-resolution remote sensing data and the CASA model to estimate carbon absorption by vegetation, offering a reliable quantification of carbon sequestration within each village-town cluster.

###### Bottom-up based carbon source model for infrastructure

A standardized method and up-to-date activity data are essential for reporting greenhouse gas emissions,^45^ and all sources of emissions related to production and consumption should be included as fully as possible in carbon emission inventories.^46^^,^^47^ At national and urban scales, the IPCC's 'Guidelines for National GHG Inventories'^48^ provide a systematic framework for quantifying emissions from major sources, such as energy, industrial processes, agriculture, and waste. To balance data accessibility and technical precision, regions typically develop tailored frameworks and models specific to their local contexts.^49^

At the village scale, emission accounting faces unique challenges, primarily due to the lack of centralized data, necessitating a bottom-up approach for accurate carbon accounting.^50^^,^^51^ Accurate village-level infrastructure carbon accounting requires detailed field surveys and specialized databases, particularly for infrastructure energy use calculations. In this study, grey infrastructure includes residential buildings (such as houses and apartment complexes), public facilities (e.g., schools, hospitals, administrative offices), and transportation infrastructure (e.g., roads, streetlights, traffic systems). Their energy consumption—derived from heating, electricity usage, fuel for transportation, and lighting—is incorporated into the carbon accounting framework to reflect the operational carbon footprint of village infrastructure. This includes emissions from both direct fuel combustion and indirect electricity consumption.

This study focuses on carbon emissions from the operational stage of infrastructure (Scope 1& 2) and excludes embodied emissions (Scope 3), which are associated with material and equipment production. This boundary setting ensures feasibility within the constraints of limited data resources, while focusing on the major emission sources. Using a bottom-up data collection approach, we revised an operational carbon emission calculation model and employed the Emission Coefficient Method (ECM) as outlined in the 2006 IPCC Guidelines for National GHG Inventories. The emission factor for electricity was modified based on the baseline coefficients published by China’s National Development and Reform Commission, ensuring alignment with the local energy structure and actual conditions.^52^ By this approach, we accurately quantify infrastructure emissions at the village scale, providing a scientific basis for formulating mitigation policies tailored to rural areas. The formula is as follows:

(Equation 1) $$CE=\sum_{i=1}^{n}\left(Q_{i}\times EF_{i}\right)$$

where *CE* is the total CO_2_ emissions from all grey infrastructure in the village (kg); i represents the i^th^ type of energy used; $Q_{i}$ is the consumption of different energy types, converted to standard coal equivalents (tce); ${EF}_{i}$ is the carbon emission factor for the i^th^ energy type (kg/tce). Carbon emission factors for various energy types are provided in Table S2.

###### Top-down based carbon sink model for infrastructure

The sample plot inventory method,^53^ the IPCC inventory method,^54^ and the model simulation methods^55^ are examples of methods for estimating carbon sinks. It is possible to obtain high-precision point source data using the sample plot survey method, however, its overall accuracy depends on the sampling methods and is time consuming and may result in errors. In villages with green infrastructure with high spatial heterogeneity and complex vegetation structures, the sample plot method is prohibitively labour-intensive. While the IPCC inventory method is straightforward, it lacks biophysical mechanisms, causing it to be inaccurate. In addition to improving model simulation accuracy through improved remote sensing data precision, advances in remote sensing and GIS technologies provide foundational support for carbon sink accounting and monitoring.^56^ Net Ecosystem Productivity (NEP, g C·m^−2^·a^−1^) is a key indicator that directly describes the carbon source/sink capacity of terrestrial ecosystems,^57^^,^^58^ defined as the difference between NPP (g C·m^−2^·a^−1^) and heterotrophic respiration (*R*_*H*_, g C·m^−2^·a^−1^):

(Equation 2) $$NEP=NPP-R_{H}$$

As a representative light-use efficiency model,^16^ CASA was selected because it is available, considers carbon cycle mechanisms, requires fewer physical parameters, and has relatively small errors. By combining high-resolution remote sensing data with vegetation cover and light utilization in village-town clusters, CASA accurately reflects the vegetation cover and light utilization of villages. We estimate NPP using CASA and derive vegetation carbon absorption by integrating a biomass-carbon sink model (see Figure S2).

The main parameters of the CASA model include Absorbed Photosynthetically Active Radiation (*APAR*) and actual light-use efficiency (*ε*)^59^:

(Equation 3) NPP(x,t)=APAR(x,t)×ε(x,t)

where, *NPP*(*x, t*) is NPP at pixel *x* in month *t* (g C·m^-2^). *APAR*(*x, t*) is APAR at pixel *x* in month *t* (MJ·m^-2^); ε(x,t) is ε at pixel *x* in month *t* (gC·MJ^-1^). Following the terrestrial vegetation NPP remote sensing estimation methods, we calculate *APAR* and *ε* using solar radiation, precipitation, temperature, vegetation type, and high-resolution Sentinel-2 & Landsat image data:

(Equation 4) APAR(x,t)=SOL(x,t)×FPAR(x,t)×0.5

(Equation 5) $$FPAR\left(x,t\right)=\alpha FPAR_{NDVI}+\left(1-\alpha \right)FPAR_{SR}$$

(Equation 6) $$FPAR_{NDVI}=\frac{NDVI\left(x,t\right)-NDVI\left(i,\min\right)}{NDVI\left(i,\max\right)-NDVI\left(i,\min\right)}\times \left({FPAR}_{\max}-{FPAR}_{\min}\right)+{FPAR}_{\min}$$

(Equation 7) $$FPAR_{SR}=\frac{SR\left(x,t\right)-SR\left(i,\min\right)}{SR\left(i,\max\right)-SR\left(i,\min\right)}\times \left({FPAR}_{\max}-{FPAR}_{\min}\right)+{FPAR}_{\min}$$

(Equation 8) $$SR\left(x,t\right)=\frac{1+NDVI\left(x,t\right)}{1-NDVI\left(x,t\right)}$$

(Equation 9) $$\varepsilon \left(x,t\right)=T_{\varepsilon 1}\left(x,t\right)\times T_{\varepsilon 2}\left(x,t\right)\times W_{\varepsilon }\left(x,t\right)\times \varepsilon_{\max}$$

where, *SOL* (*x*, *t*) is the total solar radiation at pixel *x* in month *t* (MJ·m^−2^·month^−1^); The constant 0.5 represents the proportion of total solar radiation usable by vegetation as photosynthetically active radiation; *NDVI* (*i*, max) and *NDVI* (*i*, min) are the maximum and minimum NDVI values for vegetation type *i*; *FPAR*_min_ and *FPAR*_max_ are the minimum and maximum *FPAR* values (0.001 and 0.950, respectively). *SR* (*i,* min) and *SR* (*i,* max) correspond to the 5th and 95th percentile NDVI values for vegetation type *i* respectively; *α* is set to 0.5.^60^ *T*_*ε*1_ (*x*, *t*) and *T*_*ε*2_ (*x*, *t*) represent the stress effects of low and high temperatures on light-use efficiency; *W*_ε_ (*x*, *t*) is the water stress coefficient; *ε*_max_ is the maximum light-use efficiency.

Based on photosynthesis principles, NPP represents the remaining organic matter after subtracting autotrophic respiration from the total produced via photosynthesis per unit time and area. Using the heterotrophic respiration model established by,^61^ we estimate *R*_*H*_ through a regression equation relating temperature, precipitation, and carbon emissions:

(Equation 10) $$R_{H}=0.22\times \left(\exp\left(0.0913T\right)+\ln\left(0.3145R+1\right)\right)\times 30\times 46.5\%$$

where, *T* is temperature (°C), *R* is precipitation (mm). To quantitatively analyse the spatial distribution of carbon emissions within the study area and to investigate their potential influencing factors, we derive the net carbon emission intensity (NCEI, tCO_2_e·km^-2^·a^-1^) as an indicator. This indicator integrates carbon emissions and absorption rates, which are calculated using the following set of equations:

(Equation 11) $$CSI=\frac{NEP\times \frac{M_{co_{2}}}{M_{c}}}{A_{land}}$$

(Equation 12) $$CEI=\frac{CE}{A_{land}}$$

(Equation 13) NCEI=CEI-CSI

where, *M*_*CO2*_/*M*_*C*_ is molecular mass ratio of CO_2_ to carbon (44/12). *A*_*land*_ is land area of the study region. *CSI* is carbon absorption per unit area, measured in CO_2_ equivalents. *CEI* is carbon emission per unit area.

##### Quantifying integrated green-grey infrastructure land cover patterns

Rather than focusing on individual infrastructure types, this study investigates the broader concept of green and grey infrastructure, emphasizing their roles in shaping carbon emissions. Recognizing that IGGI-LCP influences the dependent variable (NCEI) through multiple pathways—such as spatial coverage, compositional structure, and interactive effects—we derived independent variables from multi-source datasets and employed a comprehensive set of green and grey land cover indicators (Table S3).

The Green Coverage Ratio (G) is represented by NDVI, a widely used vegetation index strongly correlated with vegetation productivity and carbon sequestration potential.^62^^,^^63^ NDVI reflects vegetation health and density, which are critical for carbon absorption and emission dynamics. Compared to land classification methods, NDVI captures the functional role of vegetation in carbon cycling, aligning well with studies on carbon dynamics. To further capture the ecological integrity of the region, the Ecological Core Area Percentage (R_core_) is introduced, representing the proportion of core ecological zones within the study area and highlighting the importance of ecologically significant patches. Similarly, the Ecological Edge-Core Percentage (R_edge_) quantifies ecological fragmentation by assessing the ratio of edge areas to core areas, providing a robust indicator of ecosystem quality grounded in landscape ecology principles.^64^ On the other hand, the Grey Coverage Ratio (V) measures the proportion of land area directly occupied by grey infrastructure, including buildings, roads, and bridges. This metric reflects the physical footprint of human development on the landscape and has been shown to significantly correlate with carbon emissions.^65^ Conceptually, while development intensity broadly captures the concentration of human activity and land use, the Grey Coverage Ratio specifically quantifies the area physically occupied by grey infrastructure, offering a focused perspective on its spatial impact. As such, it serves as a critical indicator of the environmental implications of human development, particularly its influence on carbon dynamics. To further assess the spatial efficiency of grey infrastructure, we compute the Grey Infrastructure Space Utilization Rate (V_util_), which incorporates both vertical and horizontal utilization dimensions. This metric provides insight into the efficiency of grey infrastructure deployment within the landscape and its potential implications for carbon emissions and broader environmental sustainability.

In this study, the Normalized Difference Green-Grey Index (NDGG) is utilized as an interactive indicator to evaluate the spatial balance and relative dominance of green and grey land cover. Considering the methodological differences in quantifying these two metrics—where green coverage is represented as a relative measure and grey coverage as an absolute proportion—NDGG normalizes them to ensure comparability. Unlike traditional interaction terms that simply combine variables additively or multiplicatively,^66^ NDGG uses a normalized difference to reflect the spatial interplay between green and grey land cover.

NDGG values range from -1 to 1. Values closer to 1 indicate a dominance of grey infrastructure land cover, meaning that artificial structures such as buildings and impervious surfaces account for a larger proportion of the land cover. Conversely, values closer to -1 signify a predominance of green land cover, where ecological vegetation constitutes a greater share. An NDGG value of 0 represents a balanced spatial distribution, indicating equilibrium between green and grey land cover. The NDGG concept draws on the broader family of normalized difference indices widely used in remote sensing, such as NDVI, and parallels the Normalized Difference Green-Grey Volume Index derived from LiDAR studies (e.g., Rome, Italy). Those studies reported strong correlations between three-dimensional NDGG and NDVI (0.76–0.83) and strong negative correlations with impervious surface ratios (−0.75 – −0.83), providing empirical support for our two-dimensional adaptation. Furthermore, NDGG bridges the gap between Normalized Difference Green-Building Volume (NDGB) (used to link green volume and building volume to human health perceptions) and the more generalized Grey-Green Scale (GGSCI) used at regional scales. While our NDGG index does not capture vertical heterogeneity due to the lack of LiDAR data, it remains a useful proxy for green-grey balance when integrated with socioeconomic mediators (D_GDP_, D_pop_).^67^

To determine operationally robust green–grey balance thresholds, we conducted a data-driven but policy-oriented procedure (Figure 4). First, we stabilised variance by transforming NCEI using $y_{s}=\mathrm{asinh}\;\left(y/1000\right)$, accommodating negative values and compressing extreme positive values. We then fitted a continuous three-segment (“hinge”) regression model:

(Equation 14) $$y_{s}=\beta_{0}+\beta_{1}X_{1}+\beta_{2}{\left(x-c_{1}\right)}_{+}+\beta_{3}{\left(x-c_{2}\right)}_{+}+\epsilon$$

where *x* is NDGG and (·)_+_ denotes the positive part. A grid search over candidate breakpoints ($c_{1}$, $c_{2}$) on [–0.9, +0.9] (minimum separation 0.15) minimised the residual sum of squares. Bootstrapping (200 replicates) yielded mean breakpoints ${\bar{c}}_{1}$ ≈ −0.489 and ${\bar{c}}_{2}$ ≈ +0.076 with 95% confidence intervals [–0.811, –0.087] and [–0.541, +0.781]. These data-driven breakpoints indicate where the relationship between NDGG and NCEI changes, but the first breakpoint (≈ −0.489) is far below zero and the second (≈ +0.076) is close to zero, and both carry wide confidence intervals. To translate these results into a zoning rule, we examined the distribution of NDGG values and considered a range of candidate thresholds. We sought a pair of cut-offs that (i) fell within the confidence bands of the breakpoints, (ii) lay in the region where observations are plentiful and the NDGG–NCEI relationship is stable, and (iii) maintained symmetry around zero in keeping with the NDGG index (positive values denote grey dominance, negative values denote green dominance). Through this process the ±0.20 values emerged as a reasonable compromise: they reside comfortably within the 95 % confidence intervals of both breakpoints, coincide with the densest portion of the NDGG distribution (approximately –0.3 to +0.3) and provide a clear, balanced distinction between green-dominant and grey-dominant conditions. We also note that ±0.2 thresholds are widely used in remote-sensing indices such as NDVI to delineate vegetated versus non-vegetated surfaces, offering an intuitive analogue for planners. Because the NDGG > 0.20 region contains only four observations with high variance, we summarised the central trend there using binned medians and a LOWESS smoother rather than imposing a third linear segment. For binary classification of net sources versus net sinks, a ROC analysis yielded an optimal threshold of –0.587 and an AUC of 0.909; this asymmetrical threshold is suited to classification tasks but not to spatial planning. We therefore propose –0.20 ≤ NDGG ≤ +0.20 as the balanced green–grey range. This band was derived post hoc from the data and is intended to provide a simple, symmetric and interpretable guide for low-carbon planning.

###### Ordinary least-squares regression (OLS)

The OLS model is a traditional global regression method widely used in urban planning to identify the relationship between a dependent variable and its influencing factors.^68^^,^^69^ We employed the OLS model to determine the relationship between NCEI and IGGI-LCP and served it as the base model. The form of OLS is expressed as:

(Equation 15) $$Y=\beta_{0}+\beta_{1}X_{1}+\beta_{2}X_{2}+\cdots +\beta_{n}X_{n}+\zeta$$

where *Y* is the dependent variable (*NCEI*), *X*_1_, *X*_2_ …*X*_n_ are the explanatory variables representing IGGI-LCP indicators, β are the estimated coefficients, and ζ is the random error.

While the OLS model provides an optimal unbiased estimation, it assumes that the relationship between variables is constant across the study area, ignoring spatial non-stationarity.^70^ According to Tobler's First Law of Geography, “everything is related to everything else, but near things are more related than distant things.” The relationships between variables may change due to geographic location, environmental factors, and other spatial characteristics. This suggests that the OLS model may not be well-suited to accurately capture the true relationships between variables,^71^ as it overlooks the spatial heterogeneity inherent in geographic phenomena. Therefore, we adopted models that account for spatial variation to better capture the relationships in our data.

###### Geographically weighted regression (GWR)

Compared to the OLS model, GWR is a local linear regression model where parameter estimation vary across the spatial domain.^72^ This approach fully considers spatial non-stationarity and location dependence by building non-parametric models in different regions,^73^ thereby enhancing the scientific validity and effectiveness of the model in geographic studies. The mathematical form of the GWR model is:

(Equation 16) $$y_{i}=\beta_{0}\left(u_{i},v_{i}\right)+\sum_{j=1}^{k}\beta_{j}\left(u_{i},v_{i}\right)X_{ij}+\varepsilon_{i}$$

where $y_{i}$ is dependent variable at location *i*; $X_{ij}$ are the *j*^th^ explanatory variable at location *i*; $\beta_{0}$ and $\beta_{j}$ are the estimated coefficient at location *i*; ($u_{i},v_{i}$) are the coordinates of location *i*; $\varepsilon_{i}$ is the random error at location *i*. The core of the GWR model is the weight matrix, which represents the spatial relationship between the focal point and nearby samples. Several kernel functions, such as Gaussian, bi-square, and tri-cube functions, are commonly used to define the weight matrix.^70^^,^^71^ In this paper, we selected the Gaussian function to determine spatial weights, as it is a continuous, monotonic decreasing function of distance and provides sufficient local observations to calibrate the model. The Gaussian function is expressed as:

(Equation 17) $$W_{ij}=\exp\left[-\frac{1}{2}{\left(\frac{d_{ij}}{b}\right)}^{2}\right]$$

where, *d*_*ij*_ is the distance between locations *i* and *j*; *b* is bandwidth. A larger bandwidth represents a broader influence range and a slower decay of weights with distance. To mitigate risks of overfitting in sparse sample areas, we adopt a Gaussian kernel function with adaptive bandwidth, which adjusts the bandwidth dynamically to accommodate variations in sample size and data density.^74^

###### Multiscale geographically weighted regression (MGWR)

While GWR applies a single bandwidth for all variables—potentially introducing bias and noise—MGWR improves upon this by considering scale effects and adaptively adjusting bandwidths for different variables.^75^^,^^76^^,^^77^ This allows each independent variable to have its own bandwidth, enhancing the accuracy of spatial data representation and better explaining the spatial influence of independent variable coefficients. The specific function of the model is as follows:

(Equation 18) $$y_{i}=\sum_{j=0}^{q}\beta_{j}X_{ij}+\sum_{j=q+1}^{p}\beta_{j}\left(u_{i},v_{i}\right)X_{ij}+\varepsilon_{i}$$

where $y_{i}$ is the dependent variable; $X_{ij}$ are the *j*^th^ explanatory variables at location *i*; $\beta_{j}\left(u_{i},v_{i}\right)$ are the estimated coefficients of local variables; $\beta_{j}\left(j=0,1,2...q\right)$ are the estimated coefficients of local variables; $\left(u_{i},v_{i}\right)$ are the coordinates of location *i*; $\varepsilon_{i}$ is the random error at location *i*. A key component of the MGWR model is the selection of accurate and reliable methods for separating global from local variables.^70^^,^^73^

###### Spatial autocorrelation and variable selection

Spatial autocorrelation analysis is a crucial step in spatial modeling, used to detect the correlation between variables and their proximity in geographical space.^78^^,^^79^ In this study, we investigated spatial autocorrelation using Global Moran's I, calculated in ArcGIS 10.5. The formula for Moran's I is:

(Equation 19) $$I=\frac{n\sum_{i=1}^{n}\sum_{j=1}^{n}w_{ij}\left(x_{i}-\bar{x}\right)\left(x_{j}-\bar{x}\right)}{\sum_{i=1}^{n}\sum_{j=1}^{n}w_{ij}\sum_{i=1}^{n}{\left(x_{i}-\bar{x}\right)}^{2}}$$

where n is the number of geographic units; $x_{i}$ and $x_{j}$ are the values at locations *i* and *j*; $w_{ij}$ is the spatial weight between locations *i* and *j*; Moran’s I value range from -1 to 1, and close to 0 indicates no spatial autocorrelation.^78^ A positive value suggests spatial clustering of similar values, while a negative value indicates dispersion. To evaluate the null hypothesis of no spatial autocorrelation, we calculated the z-score:

(Equation 20) $$Z=\frac{I-E\left(I\right)}{\sqrt{\text{var}\left(I\right)}}$$

if |Z|>1.96, the original null hypothesis can be rejected at the 0.05 significance level, indicating that Moran's I is statistically significant. A positive z-score indicates that the variable is spatially clustered with high or low values.

In addition, Pearson's correlation coefficient test was used to detect multicollinearity among the study variables. And any variable with a coefficient greater than 0.7 should be excluded from models as it indicates strong correlations.^80^ The results are shown in Figure S3. In this study, R_core_ was retained over R_edge_ due to their high negative correlation (r = −0.73) and R_core_’s stronger link to NCEI (r = −0.60), reflecting the critical role of ecological core areas in carbon absorption. NDGG was selected instead of G and V as it is a composite index derived from these variables, effectively capturing the grey-green balance with strong correlations to both (r = 0.85 and r = −0.81), avoiding redundancy. The retained variables (NDGG, R_core_, V_util_, D_GDP_, D_pop_) all showed significant correlations with NCEI (r > 0.30, p < 0.01), ensuring their relevance for explaining spatial and socioeconomic variations in carbon emissions.

After the above step, we generated the specific models as follows. Firstly, we preprocessed the model data, including valid data detection, abnormal data elimination, missing data correction and normality analysis of selected variables. As shown in Figure S4, all variables exhibited varying degrees of non-normal distribution, reflecting the inherent non-stationarity of village-level data. To address this, we applied natural logarithmic transformations to V_util_, D_GDP_, and D_pop_, resulting in ln(V_util_), ln(D_GDP_) and ln(D_pop_), while retaining R_core_, NDGG, and NCEI in their original forms to preserve interpretive clarity and data fidelity. Specifically, given the inherent variability and heterogeneity of village-level data, R_core_ closely approximates a normal distribution, making a logarithmic transformation unnecessary. While NDGG and NCEI contain negative values, and a log transformation could theoretically be applied by shifting the data upward, such adjustments risk distorting the data's inherent structure and introducing artificial biases. Retaining these variables in their original forms ensures the authenticity of the data, maintains its statistical characteristics, and preserves its real-world interpretability.

Using these pre-processed variables, we developed three stepwise OLS regression models: OLS 1 included ln(V_util_) and R_core_, establishing a baseline; OLS 2 added NDGG to capture green grey interactions; and OLS 3 incorporated ln(D_GDP_) and ln(D_pop_), introducing economic and population density effects. This stepwise approach allowed us to systematically examine the incremental contributions of variables while ensuring the logical coherence and statistical soundness of the models.

### Quantification and statistical analysis

All analyses in this study were descriptive or spatial-regression based, and no inter-group hypothesis tests (e.g., t-tests or ANOVA) were performed. The primary unit of analysis was the village (*n* = 74), and the exact value of *n* and its meaning are indicated in the figure legends. Continuous variables, such as net carbon emission intensity and the Normalized Difference Green-Grey Index, were summarised using medians and interquartile ranges (IQR) or other appropriate descriptive statistics; measures of central tendency and dispersion (e.g., medians, IQRs and 95 % confidence intervals) are reported in the tables and figure legends. Relationships between land-cover patterns and emissions were modelled using stepwise ordinary least squares (OLS), Geographically Weighted Regression (GWR) and Multiscale GWR (MGWR) to capture both global and local effects of grey infrastructure utilisation (V_util_), ecological core area (R_core_), NDGG and socioeconomic factors [economic density (D_GDP_) and population density (D_pop_)]. Threshold analyses and predictive performance assessments—including hinge-type piecewise regression, cross-validation and Monte Carlo simulations—were performed to identify NDGG breakpoints and quantify uncertainty. Data processing and analyses were conducted using ArcGIS 10.5 and the *mgwr* Python package. Detailed statistical parameters and additional data are provided in the figure legends, tables and results section of the manuscript, and supplementary figures and data tables are presented in Supplementary Documents S1.

### Additional resources

This study did not generate additional resources.
