# Supplementary material for: Balancing integrated green-grey infrastructure shapes carbon emissions in village-town clusters
Source: iScience. 2025 Oct 30;28(12):113902. doi: 10.1016/j.isci.2025.113902 (PMC12702223; doi:10.1016/j.isci.2025.113902)
Supplement: Document S1. Figures S1–S4 and Tables S1–S3 [file mmc1.pdf]

iScience, Volume *28*

Supplemental information

Balancing integrated green-grey infrastructure shapes carbon emissions in village-town clusters

Zhuo-Yang Sun and Xiaoqing Zhu

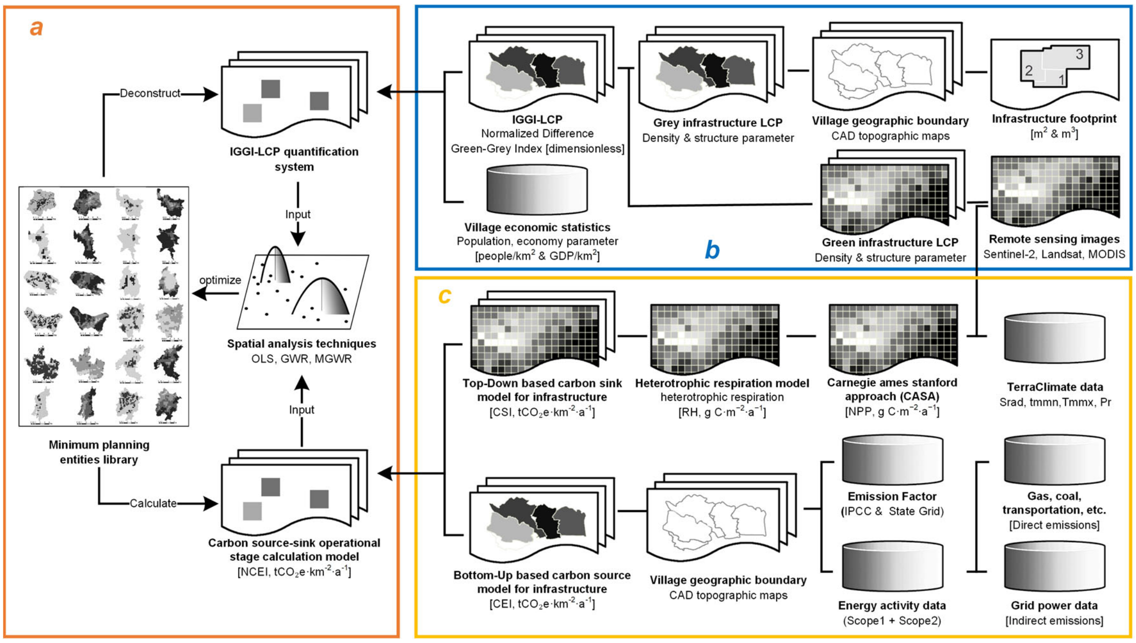

Figure S1. Methodological framework for data integration, IGGI-LCP indicator computation, and operational carbon source–sink estimation. a. Data Integration and Spatial Regression: Integration of multiple datasets and application of spatial regression methods, with results fed back to village-town clusters; **b.** IGGI-LCP Indicator system: Quantification of Integrated green-grey infrastructure land cover patterns; **c.** Carbon Source-Sink Operational Stage Calculation Model: Calculation of carbon emissions during the operational stage of infrastructure.

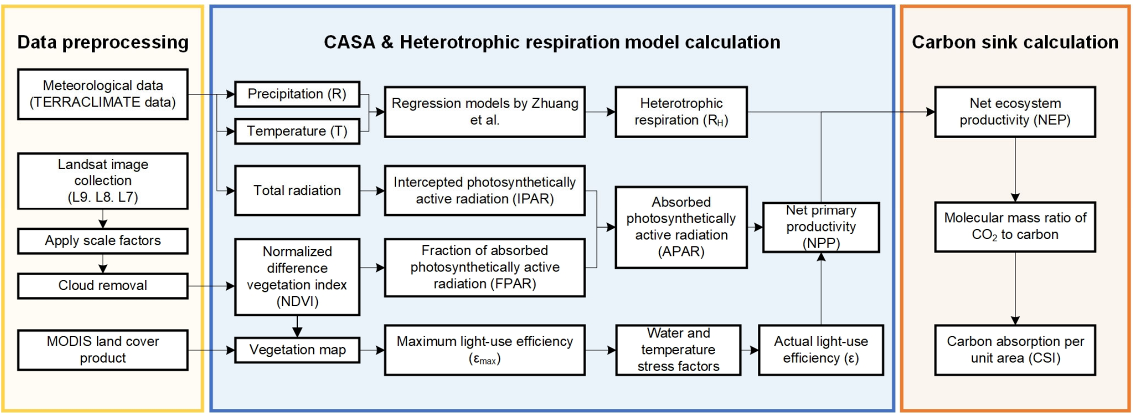

Figure S2. Carbon sink estimation framework using the CASA model.

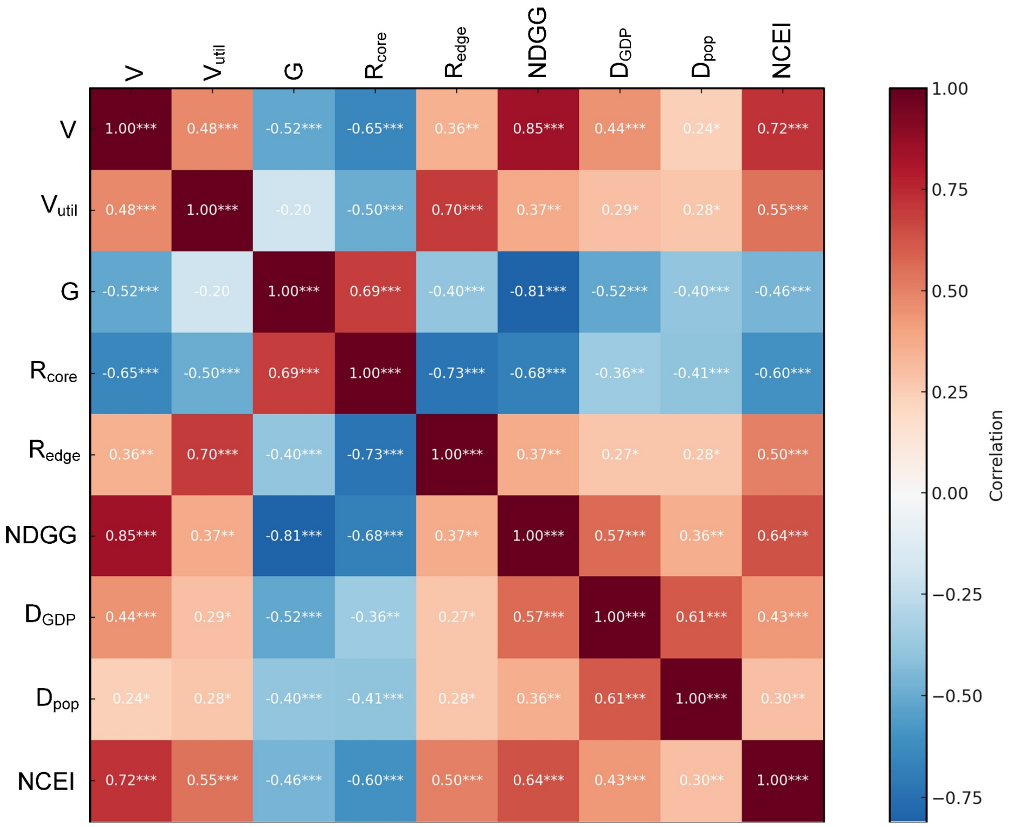

Figure S3. Pearson correlation matrix of IGGI-LCP variables and explanatory factors. ***Note:*** *The correlation matrix displays Pearson correlation coefficients, categorized as follows:* $|r| > 0.7$ *indicates a strong linear relationship,* $0.3 \leq |r| \leq 0.7$ *indicates a moderate linear relationship, and* $|r| < 0.3$ *suggests a weak or negligible linear relationship. Statistical significance levels are marked by asterisks:* ***$p < 0.001$, **$p < 0.01$, *$p < 0.05$.

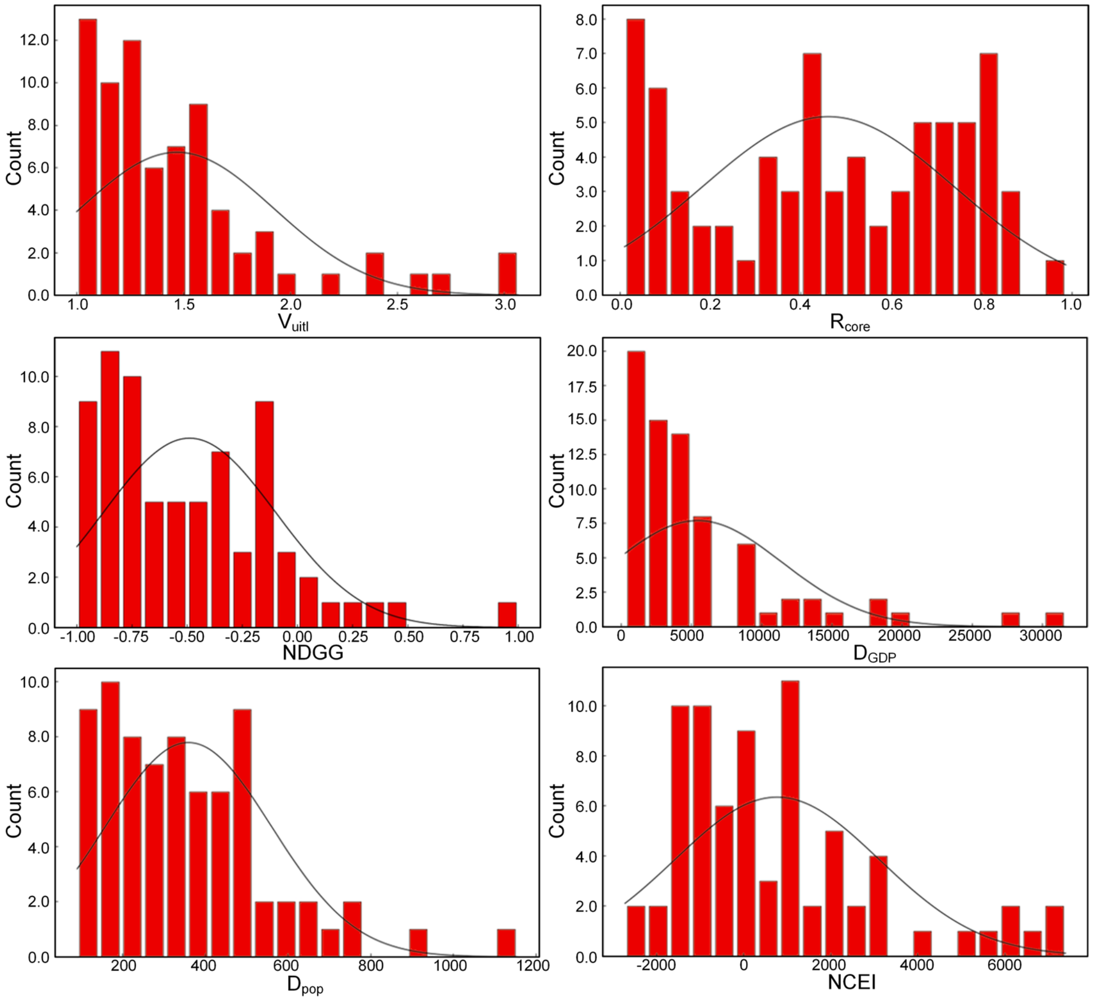

Figure S4. Distributions of net carbon emission intensity and IGGI-LCP indicators.

Table S1. Data sources and processing methods used in this study.

	Data Name	Data Source	Spatial/Temporal Resolution	Processing Method
Infrastructure Footprint Data	Township CAD Topographic Maps (China Geodetic Coordinate System 2000)	Township Government	1:500 scale	Digitized CAD format, using GIS tools for integration
	Sentinel-2 (COPERNICUS/S2_SR_HARMONIZED)	European Space Agency (ESA)	10m (spatial), 5-day revisit	Core dataset for NDVI calculations
	Landsat (9, 8 OLI and TIRS, 7)	United States Geological Survey (USGS)	30m (spatial), 16-day revisit	Supplementary NDVI calculations for validation or gap-filling where Sentinel-

				2 data was not available
Carbon source Data	Electric Power	State Grid Corporation	Aggregated yearly data	Field data collection and sampling
	Coal			
	Firewood			
	Gasoline	Sampling Investigation from 74 villages	Survey data	Converted from local consumption data
	Diesel			
	Coal Gas			
	Natural Gas			
	Travel Mileage per Year of Buses among Villages	Bus Company	Average annual mileage	Sampled from fleet management data
	Private Car (Average Annual Mileage)			
	Motorcycle (Average Annual Mileage)	Sampling Investigation	Survey data	Calculated from survey data
	Truck (Average Annual Mileage)			
Carbon sink Data	Sentinel-2 (COPERNICUS/S2_SR_HARMONIZED)	European Space Agency (ESA)	10m (spatial), 5-day revisit	Core dataset for NDVI calculations
	TerraClimate Global Monthly Climate Dataset (IDAHO_EPSCOR/TERRACLIMATE)	University of California Merced	4 km (spatial), monthly	Climate variables for NPP estimation (CASA model)
	MODIS Vegetation Cover Type (MOD12Q1)	United States Geological Survey (USGS)	500m (spatial), annual	Vegetation cover classification for NPP estimation
Socioeconomic Data	GDP Statistical Data of Villages and Towns	Village Committee (Statistics of the Basic Situation of Village Economy)	Aggregated annual data	GDP density calculated based on village/town area
	Population Statistical Data of Villages and Towns	Village Committee (Village Population Census Table)	Aggregated annual data	Population density calculated based on village/town area

Table S2. Carbon Emission Factors for Various Energy Types

Reference	Energy Type	Carbon Emission Factor	Unit
China's National Development and Reform Commission	Electricity	0.81×10^{-3}	tCO_2/kWh
2006 IPCC Guidelines for National GHG Inventories	Coal	2.68	tCO_2/tce
	Firewood	1.44	
	Gasoline	2.99	
	Diesel	3.16	$tCO_2/10^4\ m^3$
	Coal Gas	8.55	
	Natural Gas	8.55	

Table S3. Variables of Integrated green-grey infrastructure Land Cover Patterns

Type		Variable	Formula	Definition
Independent variable	Green infrastructure density & structure parameter	Green Coverage Ratio (G) [dimensionless]	$G=\frac{(NIR-RED)}{(NIR+RED)}$	where NIR is near-infrared reflectance, and RED is red light reflectance. Represents the NDVI, indicating the growth and coverage of vegetation.
		Ecological Core Area Percentage (R_{core}) [%]	$R_{core}=\left(\frac{\sum_{i=1}^{n} Pixels_{core,i}}{\sum_{j=1}^{i} Pixels_{land,j}}\right)\times100\%$	Where $\sum_{i=1}^{n} Pixels_{core,i}$ is the sum of ecological core pixels, and $\sum_{j=1}^{i} Pixels_{land,j}$ is the sum of land pixels in the study area.
		Ecological Edge-Core Percentage (R_{edge}) [%]	$R_{edge}=\left(\frac{\sum_{k=1}^{p} Pixels_{edge,k}}{\sum_{i=1}^{n} Pixels_{core,i}}\right)\times100\%$	Where $\sum_{k=1}^{p} Pixels_{edge,k}$ is the sum of ecological edge pixels, and $\sum_{i=1}^{n} Pixels_{core,i}$ is the sum of ecological core pixels.

	Grey infrastructure density & structure parameter	Grey Coverage Ratio (V) [dimensionless]	$V=\frac{\sum A_{grey}}{A_{land}}$	Where A_{grey} is the area occupied by all grey infrastructures (including buildings, roads, etc.) in the region, and A_{land} is the land area of the study region.
		Grey Infrastructure Space Utilization Rate (V_{util}) [%]	$V_{util}=\left(\frac{\sum_{i=1}^{n} L_i \times A_{grey,i}}{A_{land}}\right)\times 100\%$	Where L_i is the number of floors of the ith grey infrastructure, $A_{grey,i}$ is the area per floor of the ith grey infrastructure, and A_{land} is the land area of the study region.
	Integrated green-grey infrastructure parameter	Normalized Difference Green-Grey Index ($NDGG$) [dimensionless]	$NDGG=\frac{(V-G)}{(V+G)}$	Where V is the grey coverage ratio, and G is the green coverage ratio.
Covariate	Population	Population Density (D_{pop}) [people/km^2]	$D_{pop}=\frac{P_{total}}{A_{land}}$	Where P_{total} is the total population within the study area, and A_{land} is the land area of the study region.
	Economy	GDP Density (D_{GDP}) [GDP/km^2]	$D_{GDP}=\frac{GDP_{total}}{A_{land}}$	Where GDP_{total} is the total Gross Domestic Product within the study area, and A_{land} is the land area of the study region.

Dependent variable	Carbon emission	Net Carbon Emission Intensity (*NCEI*) [$tCO_2e \cdot km^{-2} \cdot a^{-1}$]	$NCEI = CEI - CSI$	where *CSI* is the carbon absorption per unit area, measured in CO_2 equivalents; *CEI* is the carbon emission per unit area.
